# Beyond the sandy bottom: evolutionary and taxonomic insights into lizardfishes (Teleostei: Aulopiformes)

**DOI:** 10.7717/peerj.20735

**Published:** 2026-03-06

**Authors:** Shih-Yu Wang, Hsin Lee, Barry C. Russell, Wei-Jen Chen

**Affiliations:** 1 Institute of Oceanography, National Taiwan University, Taipei, Taiwan; 2 Department of Exhibition, National Museum of Marine Biology and Aquarium, Pingtung, Taiwan; 3 Museum & Art Gallery of the Northern Territory, Darwin, Northern Territory, Australia

**Keywords:** Synodontidae, Harpadontidae, Phylogeny, Species delimitation, Cryptic diversity, Multi-gene analysis, Systematics, Indo-West Pacific, Deep sea, New family

## Abstract

**Background:**

Lizardfishes (family Synodontidae) are benthic, carnivorous fishes that primarily inhabit tropical and subtropical sandy seabeds and play an important role in benthic ecosystem functioning. They are characterized by cylindrical bodies and large mouths and currently comprise 84 recognized species across four genera: *Synodus*, *Harpadon*, *Saurida*, and *Trachinocephalus*. However, the systematics of the Synodontidae—from higher-level classification to species-level taxonomy—has remained contentious since the family was established by Gill in 1861, highlighting the need for a robust phylogenetic framework for further revision. This study aimed to (1) reconstruct the phylogeny of the Synodontidae and related Aulopiformes, (2) test the monophyly of the family and its genera, and (3) assess species diversity and validity using an integrated approach.

**Methods:**

We analyzed a multi-nuclear gene dataset (*RH*, *RAG1*, *ZIC1*, and *ENC1*) and complete mitochondrial genomes to investigate higher-level phylogenetic relationships, evolutionary origins, and divergence times within the Aulopiformes. Intra-familial relationships were examined using combined mitochondrial *12S* and *COI* gene datasets, and species-level taxonomy was assessed using 1,688 *COI* sequences from public and newly generated data.

**Results:**

Higher-level phylogenetic analyses revealed that the Synodontidae is not monophyletic, however, its two subfamilies, Synodontinae and Harpadontinae, formed well-supported, distinct monophyletic groups, justifying their recognition at the family level. Among the four genera, only *Harpadon* and *Trachinocephalus* were monophyletic. Divergence time estimates suggest that the last common ancestor of the Synodontinae (=Synodontidae stat. nov.) originated in the Late Cretaceous, while the Harpadontinae (=Harpadontidae stat. nov.) emerged in the Eocene. Species delimitation based on compiled *COI* sequences, using Assemble Species by Automatic Partitioning (ASAP) and Bayesian Poisson Tree Processes (bPTP) methods and supported by additional evidence, identified 108 putative species among approximately 60 morphospecies, revealing substantial cryptic diversity.

**Conclusions:**

This study clarifies the evolutionary history and taxonomy of the Synodontidae, supporting the reclassification of its subfamilies as distinct families and revealing extensive hidden species diversity. These findings provide a robust framework for future research on the systematics of lizardfishes and other aulopiform fishes.

## Introduction

The aulopiform family Synodontidae (Teleostei), known as lizardfishes, comprises 84 currently recognized species in four genera that are classified into two subfamilies. The subfamily Synodontinae includes two genera, *Synodus* Scopoli, 1777 (Scopoli, 1777) (47 species) and *Trachinocephalus* Gill, 1861 (four species) while the subfamily Harpadontinae includes the other two genera, *Saurida* Valenciennes, 1850 (26 species) and *Harpadon* Lesueur, 1825 (seven species) (Fricke, Eschmeyer & Fong, 2024; Fricke, Eschmeyer & Van der Laan, 2025). This family is believed to have the closest relationship to its aulopiform allies, Aulopidae and Pseudotrichonotidae, based on the most updated morphological evidence, which altogether are placed in the same aulopiform suborder, the Aulopoidei (Nelson, Grande & Wilson, 2016; Near & Thacker, 2024).

Being benthic carnivorous predators, lizardfishes are mostly found over sandy or muddy bottoms in shallow to mid waters of continental shelfs, with several species (*e.g.*, *Synodus variegatus* (Lacepède, 1803), *S. nigrotaeniatus* Allen, Erdmann & Peristiwady, 2017, and *Saurida gracilis* (Quoy & Gaimard, 1824)) occurring on coral reefs (Norman, 1935; Allen, Erdmann & Peristiwady, 2017). Some species, for example the species from the genus *Harpadon* exhibit a benthopelagic habit and live in deeper waters (Chaiyapo, 2013; Ganga, Thomas & Sukumaran, 2015), with the greatest depth reported at 801 m (*H. erythraeus* Klausewitz, 1983) (Klausewitz, 1983). Geographically, although the family Synodontidae can be found in tropical and subtropical marine waters throughout the world, there is little evidence to show that any of the species have a pattern of wide or global distribution. The blunt-nose lizardfish, *Trachinocephalus myops* (Forster, 1801), was thought to have a nearly circumtropical and subtropical distribution (Briggs, 1960). However, recent studies (Polanco, Acero & Betancur, 2016; Wang et al., 2018) based on morphological and molecular evidence, have challenged this traditional point of view, and recognized three distinct species. One of the species, *T. gauguini* Polanco, Acero & Betancur, 2016, was recently described based on the specimens collected from waters off the Marquesas Islands in the southern central Pacific. New evidence from the study of Wang et al. (2018) showed the distribution range of this species extends to Papua New Guinean waters, in the western Pacific. The other two congeneric species, *T. myops* and *T. trachinus* (Temminck & Schlegel, 1846), recognized by Polanco, Acero & Betancur (2016) are distributed in the Atlantic and the Indo-West Pacific (IWP) Oceans (except the Marquesas), respectively, and the most recently described species, *T. atrisignis* Prokofiev, 2019, occurs only in the western Indian Ocean near Socotra Island (Prokofiev, 2019). In Taiwan, 31 species of lizardfish (locally known as 狗母梭) have been recorded, including 17 species of *Synodus*, one species of *Trachinocephalus*, two species of *Harpadon*, and 11 species of *Saurida* (Shao, 2025). These species are typically caught on the sandy and muddy bottoms of Taiwan’s coastal waters. Although synodontid fishes do not play a significant role in Taiwan’s fishing industry, they hold value in a niche market, particularly for processed products such as the popular fish floss (a dried and powdered condiment made from shredded or mashed fish meat cooked in spices).

**Figure 1:**
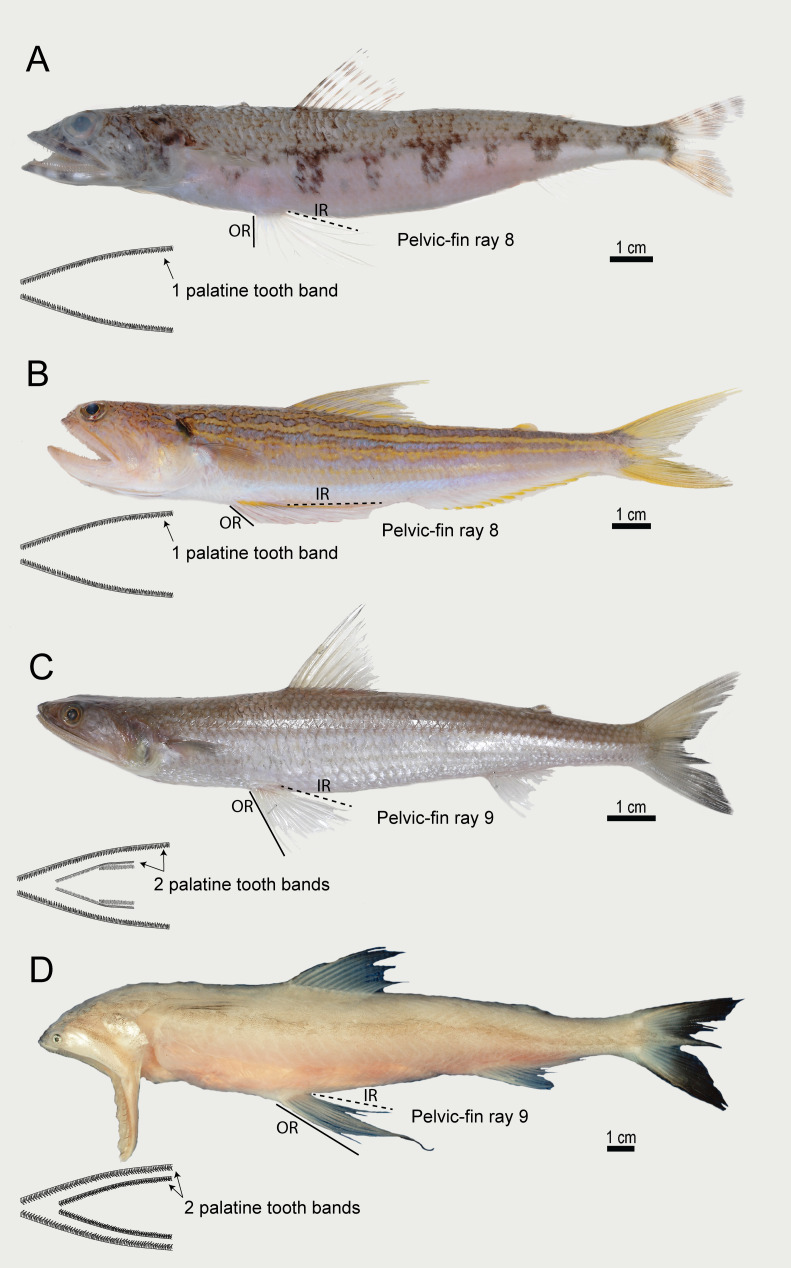
Selected species of Synodontinae (A, B) and Harpadontinae (C, D). (A) *Synodus pacificus* (sample ID: WJC10655). (B) *Trachinocephalus trachinus* (sample ID: WJC11692). (C) *Saurida micropectoralis* (sample ID: WJC9282). D, *Harpadon nehereus* (sample ID: WJC1084). OR, outermost pelvic-fin ray; IR, innermost pelvic-fin ray. This plate of fishes was kindly prepared by S.-L. Ng.

In overall appearance, lizardfishes are small to medium-sized fishes with elongated bodies (Nelson, Grande & Wilson, 2016; Norman, 1935; Russell, 2002), and the species are characterized by the following combined characters: head depressed to compressed; maxilla reduced or very slender (degenerate in species within the genus *Harpadon*); large mouth with oblique gape; laterally directed eye of small to moderate size; adipose eyelid on anterior and posterior margins of eye; branchiostegal rays 8–26; pelvic fin with eight or nine rays; dorsal fin about mid-way on back with first two rays unbranched; dorsal adipose fin usually present over base of anal fin (except *Synodus sageneus*, with a reduced or absent adipose fin); body coloration variable, but usually brightly colored for reef species and plainly colored for deeper water species (Fig. 1).

While the species of the family are similar in morphology, members of the two subfamilies can be distinguished from each other by the number of teeth bands on the palate (single band in Synodontinae, double bands in Harpadontinae), the number of pelvic fin rays (eight in Synodontinae, nine in Harpadontinae), and relative length of the inner and outer rays of the pelvic fins (inner rays of pelvic fin much longer than outer ones in Synodontinae *versus* almost equal in their length in Harpadontinae). Within the Synodontinae, the genus *Synodus* is characterized by having a snout length similar to or longer than eye diameter, while the genus *Trachinocephalus* has a relatively short snout length and a higher number of anal fin rays (15–16 rays compare to 8–15 rays in *Synodus*). The genus *Saurida* of the subfamily Harpadontinae can be further distinguished from others by the presence of scales on procurrent and primary caudal fin rays. The genus *Harpadon* is a highly specialized group with some derived characters, such as an extremely short maxilla and the absence of the ethmo-maxillary ligament, and some species lack cycloid scales on the head and body (Norman, 1935; Chaiyapo, 2013) (Fig. 1).

### Nomenclature, classification, relationships and taxonomy

The family name Synodontidae has had a somewhat confused nomenclatural history and has been applied to fishes belonging to two different orders: lizardfishes of the order Myctophiformes, and upside-down catfishes of the order Siluriformes (Dutt, 1973; Russell, 1987). Gill (1861:53) established the new family name Synodontoidae, and a new subfamily name, Synodontinae, for the lizardfish genera *Trachinocephalus* and *Synodus,* but later emended the spelling of the lizardfish family name Synodontoidae to Synodontidae (Gill, 1873). Jordan (1905) and Regan (1911a) also used the name Synodontidae for the aulopiform genera, but almost simultaneously Regan (1911b) established the identical family-group name Synodontidae, with the type-genus *Synodontis*, in the Siluriformes. Jordan (1923) subsequently established the family Mochokidae (type-genus *Mochokus* Joannis) to include *Synodontis*, and this family-group name has since been widely used for the upside-down catfishes (Van der Laan, Eschmeyer & Fricke, 2014).

A substitute family name, Synodidae, proposed for the lizardfishes by Ogilby (1910) was subsequently used by Chabanaud (1932) who mistakenly regarded Ivanova et al.’s (2007) use of the name Synodontidae as a *lapsus calami* based on *Synodontis* Cuvier rather than *Synodus* Scopoli. Berg (1940) in his *Classification of fishes both recent and fossil* recognized the family name Synodontidae only in the Siluriformes and changed the lizardfish family name to Synodidae, placing it under the order Scopeliformes. However, the name Synodidae is an incorrectly formed family-group name (Anderson, Gehringer & Berry, 1966; Sulak et al., 1985; Russell, 1987) and most modern authors (*e.g.*, Nelson, 1976; Nelson, Grande & Wilson, 2016) have used the lizardfish family name Synodontidae. Nonetheless, use of the invalid family-group name Synodidae has persisted (*e.g.*, Fowler, 1956; Smith & Smith, 1963; Golvan, 1965; Dutt, 1973; Rao, 1977; Kar & Chakraborty, 2001). We follow here the current widely accepted usage of the family-group name Synodontidae in the Aulopiformes (Van der Laan, Eschmeyer & Fricke, 2014).

**Figure 2:**
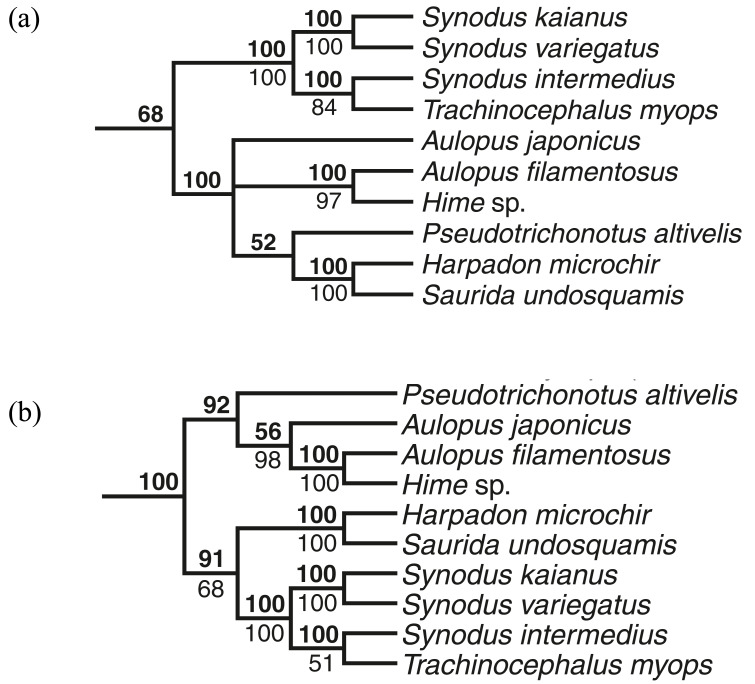
Partial phylogenetic result depicting the relationships of the synodontid taxa to the other aulopiform fishes in Davis (2010). This figure was modified from Figs. 6 and 7 of the original paper. Bayesian posterior probabilities (bold numbers above nodes) and bootstrap support values (numbers below nodes) of nodes are provided, with values below 50 omitted. (A) Results from Bayesian and Maximum Likelihood analyses of five genes: *RAG1*, *Zic1, ENC1, plagl2*, and *COI*; (B) Results from Bayesian and Maximum Likelihood analyses combining these five genes with 138 morphological characters.

Early classifications placed lizardfishes (Synodontidae) in the order “Iniomi” (Regan, 1911a), later divided into Synodontidae and Harpadontidae and allied with eight other “aulopiform”-like taxa in the superfamily Myctophoidea (Gosline, Marshall & Mead, 1966) (Fig. S1). Later, Rosen (1973) established the order Aulopiformes in which two suborders (Aulopoidei and Alepisauroidei) were recognized based on the presence of an elongated uncinate process on the second epibranchial located within the gill arches, and excluded the two myctophiform families (Myctophidae and Neoscopelidae) from the Aulopiformes (Fig. S1). Within the suborder Alepisauroidei, Rosen (1973) proposed a new superfamily Synodontoidea comprising the Synodontidae and Harpadontidae, Giganturidae and two fossil genera, †*Sardinius* and †*Volcichthys* (Fig. S1). Sulak (1977) studied osteology of the benthic “Myctophiforms” (= Aulopiformes) and considered the family Synodontidae with three different subfamilies, *i.e.,* Synodontinae (*Synodus* and *Trachinocephalus*), Harpadontinae (*Harpadon* and *Saurida*), and Bathysaurinae (*Bathysaurus*) (Fig. S1). Johnson (1982) confirmed this grouping and elevated them to family rank within the superfamily Synodontoidea. Later, Okiyama (1984), using larval evidence, argued that *Bathysaurus* larvae were too specialized to support a close relationship among bathysaurids and Synodontidae and Harpadontidae. Baldwin & Johnson (1996) analyzed 118 morphological characters and recognized four suborders of Aulopiformes with the suborder Synodontoidei included three families: Aulopidae (*Aulopus*), Pseudotrichonotidae (*Pseudotrichonotus*), and Synodontidae (*Harpadon, Saurida, Synodus,* and *Trachinocephalus*). Sato & Nakabo (2002) extended this classification by adding the family Paraulopidae (Fig. S1). Davis (2010) integrating morphological and molecular data, confirmed Synodontidae monophyly under suborder Aulopoidei (= Synodontoidei), along with Aulopidae and Pseudotrichonotidae (Fig. S1; Fig. 2B). Nelson’s “*Fishes of the World*” (Nelson, Grande & Wilson, 2016) followed Davis, recognizing the Synodontidae as a valid family with two subfamilies: Synodontinae (*Synodus* + *Trachinocephalus*), and Harpadontinae (*Saurida* + *Harpadon*). The Aulopoidei was placed with two other suborders (Paraulopoidei and Alepisauroidei) within the currently recognized Aulopiformes whose monophyly was supported by several synapomorphic characters, for example, the presence of an enlarged uncinate process on second epibranchial and the absence of swim bladder (Marshall, 1954; Gosline, Marshall & Mead, 1966; Rosen, 1973; Sulak, 1977; Johnson, 1982; Rosen, 1985; Baldwin & Johnson, 1996; Sato & Nakabo, 2002; Davis, 2010; Chaiyapo, 2013).

Rao (1977) investigated examined the osteology of Indian lizardfishes and found similar patterns among *Synodus*, *Trachinocephalus*, *Harpadon* and *Saurida*. He noted a shared traits between *Saurida* and *Harpadon* (*e.g.*, absence of orbitosphenoid, laminar expansion in the lowest pectoral radial), but a closer link between *Synodus* and *Saurida* based on vertebral characters. Hartel & Stiassny (1986) studied larval *Parasudis* (Chlorophthalmidae), also highlighted anatomical relationships with Aulopiformes, indicating that *Saurida*, *Harpadon* and *Bathysaurus* (Bathysauridae) are closely related to each other based on the absence of a “rostral cartilage”. More recently, Chaiyapo (2013) examined osteological and morphological features of 18 synodontid species and redefined the family as comprising three genera. She proposed synonymizing *Trachinocephalus* with *Synodus*, supported by synapomorphies, including the presence of tiny spines on the supraorbital and the posterior portion of the posterior process of the pelvic girdle extremely long (Chaiyapo, 2013).

The taxonomy at the species level within the family remains also problematic. Several potentially distinctive species were historically treated as a single taxon due to reliance on improper (symplesiomorphic) characters. For example, *Saurida undosquamis* (Richardson, 1848) was previously considered a widely distributed species ranging from the eastern Indian Ocean to the western Pacific, identified by the presence of dark dots on the upper margin of the caudal fin. However, subsequent research revealed that *S. undosquamis* is a species complex comprising two morpho-lineages in Japanese waters (Yamada & Ikemoto, 1979; Yamaoka, Nishiyama & Taniguchi, 1989; Yamada, 2000). Further studies by Inoue & Nakabo (2006) identified four species within this complex—*S. undosquamis*, *S. umeyoshii* (Inoue & Nakabo, 2006), *S. macrolepis* Tanaka, 1917, and *S. longimanus* Norman, 1939—based on detailed morphological evidence. More recently, a comprehensive survey of *Saurida* species in Japan identified a new species, *S. fortis* Furuhashi, Russell & Motomura, 2022, previously misidentified as *S. umeyoshii* and/or *S. wanieso* Shindo & Yamada, 1972. Morphological and genetic analyses confirmed that *S. fortis* is distinct from both species (Furuhashi, Russell & Motomura, 2023).

Another example of taxonomic confusion involves *Saurida elongata* (Temminck & Schlegel 1846), originally described from three specimens collected in Japan. The name has a long history of misapplication, resulting in significant nomenclatural ambiguity. Through re-examination of the type specimens and additional material, Russell, Motomura & Furuhashi (2025) redefined the species and resurrected *S. eso* Jordan & Herre 1907, previously synonymized with *S. elongata*. They also concluded that *S. wanieso* is a synonym of *S. elongata* and *S. microlepis* Wu & Wang, 1931, is a synonym of *S. eso* Jordan & Herre, 1907.

### Molecular Phylogenetics

Advances in molecular approaches have revolutionized fish systematics across all taxonomic levels (Chen & Mayden, 2010). By analyzing DNA sequence variation, these methods enhance species identification accuracy and facilitate the reconstruction of evolutionary relationships within target taxa (Chen & Mayden, 2010; Lo et al., 2017). Among these, mitochondrial DNA (mtDNA) has been widely employed in phylogenetic studies at intra- and inter-specific levels due to its ease of amplification, species-level variability, and compatibility with existing databases (Bargelloni et al., 2000; Bowen et al., 2001; DiBattista et al., 2013). However, mtDNA has limitations for phylogenetic inference at intergeneric or higher taxonomic levels and cannot address potential pitfalls in phylogenetic reconstruction (Schwartz & Vissing, 2002; Chen, Bonillo & Lecointre, 2003; Hurst & Jiggins, 2005; Chen et al., 2008; Chen & Mayden, 2010). To construct a robust phylogeny, it is essential to integrate data from both nuclear and mitochondrial genomes, providing a comprehensive framework for elucidating evolutionary relationships, evaluating the divergence times, and testing taxonomic hypotheses (Chen, Bonillo & Lecointre, 2003; Mayden et al., 2009).

Davis (2010) conducted the first phylogenetic analysis of the Aulopiformes, integrating both morphological and molecular evidence. His molecular dataset comprised data from four nuclear genes (*RAG1*, *ZIC1*, *ENC1*, *PLAGL2;* see material and methods for full gene names) and one mitochondrial gene (*COI*). Davis’ study produced two contrasting phylogenetic hypotheses for the Synodontidae, depending on the dataset used (Figs. 2A & 2B). Bayesian and maximum likelihood analyses based solely on the five-gene dataset suggested that the Synodontidae is non-monophyletic. In his analysis, the subfamily Harpadontinae was nested within a clade containing the Pseudotrichonotidae and Aulopidae, with this entire clade being sister to the subfamily Synodontinae (Fig. 2A). Conversely, when a total evidence approach (combining molecular and morphological data) was employed, the Synodontidae emerged as a monophyletic group, sister to a clade comprising the Pseudotrichonotidae and Aulopidae (Fig. 2B). Ultimately, Davis (2010) favored the hypothesis of a monophyletic Synodontidae based on the total evidence analysis, although only six out of the 82 currently recognized species within the family were sampled. To achieve a more comprehensive understanding of phylogeny as well as the evolutionary origin(s) of the Synodontidae, further analyses with broader taxon sampling and a more diverse set of molecular markers are desired.

In the present study, we compiled a multi-nuclear gene dataset and a mitogenome dataset to infer the high-level phylogeny of synodontids and their aulopiform allies. The nuclear gene data were mainly obtained through our own sequencing effort, while the mitogenome data were retrieved entirely from GenBank (NCBI, Nation Center for Biotechnology Information (Benson et al., 2000) (Fig. S2) and curated in MitoFish: Mitochondrial Genome Database of Fish (Iwasaki et al., 2013). Using the reconstructed phylogeny, our objectives were to (1) test the monophyly of the family, subfamilies, and their recognized genera; (2) re-examine the phylogenetic placement of the Synodontidae within the order Aulopiformes; and (3) elucidate the evolutionary relationships of synodontid fishes and their divergence times.

Additionally, we compiled a comprehensive dataset of *COI* gene sequences to assess the validity of several species, particularly those from the Indo-West Pacific (IWP), using an integrated approach in taxonomy (Puillandre et al., 2012; Hung, Russell & Chen, 2017; Lo et al., 2017; Chen & Borsa, 2020; Hurzaid et al., 2020; Lee et al., 2022). The molecular analyses included *COI* (or DNA barcoding gene) sequences generated in this study alongside publicly available data from Genbank and The Barcode of Life Data Systems (BOLD, (Ratnasingham & Hebert, 2007)), especially those recently described in (Appleyard et al., 2025). Furthermore, given the transformative role of environmental DNA (eDNA) analysis in biodiversity assessment by enabling species detection through water sampling, we also incorporated the 12S rRNA gene as a genetic marker in this study. This marker is widely utilized in fish eDNA/metabarcoding surveys due to its high specificity and extensive compatibility with reference databases (Miya et al., 2015; Miya, Gotoh & Sado, 2020). To support broader eDNA applications, we generated 12S sequences for representatives of each major lineage within the Synodontidae, identified based on the reconstructed *COI* gene tree. These sequences not only enhanced the reference database for eDNA/metabarcoding but also contributed to phylogenetic reconstructions, providing a more refined understanding of evolutionary relationships within the family.

## Materials & Methods

### Statement

This research was conducted at National Taiwan University in accordance with the university’s guidelines on animal research. As the project did not involve experiments on live animals, an ethics statement was not required. Most of the specimens examined in this study were collected during national and international expeditions authorized by the National Science and Technology Council, Taiwan, and the French National Research Agency, with permits issued by the respective authorities where applicable. The permits of New Caledonian expeditions were issued by the New Caledonia (inventory no. APA_NCPS_2016_012 and ZEE APA-NC01). The permits of BIOMAGLO were issued by the “Terres Australes et Antarctiques Françaises” (TAAF), Mayotte, and Comorres. The SAYA expedition is part of the 2e leg of the “Indian Ocean 2022” survey operated by MonacoExplorations under MoU MNHN-JMA 1176-22 (see Corbari et al., 2025). The Papua New Guinea expeditions were supported from Papua New Guinea’s National Fisheries Authority and conducted under a MoU with the University of Papua New Guinea (UPNG), and with a permit from the Papua New Guinea Department of Environment and Conservation (DEC). Portions of the text of this paper were previously published as part of a preprint of the first author’s Master’s thesis, available at https://tdr.lib.ntu.edu.tw/.

### Sample collection

A total of 244 aulopiform specimens, along with one ateleopodiform specimen (*Ateleopus japonicus* Bleeker, 1853), were collected for molecular analyses in this study. Of these, 235 specimens were from approximately 32 morphospecies of the Synodontidae (Table S1). About two-thirds of the specimens were obtained (or purchased) from local markets and fish landing sites in Taiwan, Dubai (UAE), Hainan Island (Northern South China Sea), Japan, Malaysia, Hong Kong, Philippines, Vietnam, Singapore, and Thailand. Additionally, 60 specimens were collected using French and Taiwanese research vessels during eight oceanographic cruises in localities across the IWP region—BIOMAGLO, DongSha 2014, Formosa 2022, KANACONO, Kavieng 2014, Papua Niugini, MADEEP, SAYA, and ZhongSha 2015—conducted between 2012 and 2022 as part of the “*Tropical Deep-Sea Benthos*” biodiversity exploration program and collaborative efforts between Taiwanese and French research teams. A few additional samples (∼30) were provided by collaborators (see details in “Acknowledgments” and Table S1).

The collected specimens were photographed and identified to species when possible, based on the taxonomic references (Ho, Chen & Shao, 2016; Furuhashi, Russell & Motomura, 2023; Russell, Malay & Cabebe-Barnuevo, 2024; Russell, Motomura & Furuhashi, 2025), and using morphological keys provided in the “FAO species identification guide for fishery purposes: The living marine resources of the Western Central Pacific” (Russell, 2002) and “*Fishes of Japan*” (Nakabo, 2002), with additional verification against photos available on *FishBase* (http://www.fishbase.org) (Froese & Pauly, 2024). For specimens that could not be identified to species level, especially juveniles, identification was made at the genus level first and then further confirmed using *COI* gene sequences. Methods for obtaining morphological data for identification and comparison purposes followed Anderson, Gehringer & Berry (1966). Prior to fixation in formalin, a small tissue sample (muscle or fin) was excised from each specimen, preserved in 95% ethanol, and stored at −20 °C in the Marine Biodiversity and Phylogenomics laboratory of the Institute of Oceanography, National Taiwan University, for subsequent molecular analysis. The voucher specimens were deposited in the Ichthyology collections of the National Taiwan University Museums, Taipei, Taiwan (NTUM), Academia Sinica, Taipei, Taiwan (ASIZ), National Museum of Marine Biology and Aquarium, Pingtung, Taiwan (NMMBA), Faculty of Agriculture, Kyoto University, Japan (FAKU); Museum and Art Gallery of the Northern Territory, Australia (NTM); Division of Fisheries Science, Miyazaki University, Japan (MUFS); and Kagoshima University Museum, Japan (KAUM).

### DNA extraction, PCR amplification and sequencing

Whole genomic DNA was extracted from the collected tissue samples using an automatic extractor: LabTurbo 48 Compact System (Taigen Bioscience Corporation, Piscataway, NJ, USA) with the pretreatment followed the manufacturer protocol using LabTurbo DNA Mini Kit LGD 480–500 (Taigen Bioscience Corp, Piscataway, NJ, USA). Four nuclear genes (Rhodopsin gene (*RH*), Recombination activating gene 1 (*RAG1*), gene encoding Zinc finger protein family member 1 (*zic1*), and gene encoding Ectodermal-neural cortex 1 (*ENC1*)) and three mitochondrial genes (Cytochrome oxidase subunit I gene (*COI*), one subunit of mitochondrial ribosomal RNA genes (*12S*)) were targeted and sequencing in this study. These genes were selected for their ability to provide valuable phylogenetic information according to the previous studies (Kocher et al., 1989; Chen, Bonillo & Lecointre, 2003; López, Chen & Ortí, 2004; Ward et al., 2008; Li et al., 2007; Chen et al., 2008; Davis, 2010; Chen et al., 2014; Lo et al., 2015). Primers used in this study are listed in Table S2.

The target genes were amplified using polymerase chain reaction (PCR). Temperature cycling profiles for amplification consisted of an initial denaturation stage (95 °C, 60 s for *COI*, *12S*, and 4 min for *RH*, *RAG1*, *ZIC1*, *ENC1*) followed by 35 cycles, each with a denaturation step (95 °C, 30 s for *COI*, *12S*, and 40 s for *RH*, *RAG1*, *zic1*, *ENC1*), an annealing step (51 °C, 30 s for *COI*; 55 °C, 40 s for *12S*, and *RH*; 53 °C, 40 s for *RAG1*; 57 °C, 40 s for *zic1*, 54 °C, 40 s for *ENC1*) and an elongation step (72 °C, 40 s for *COI*, *12S*, 75 s for *RH*, *zic1*, *ENC1*, and 90 s for *RAG1*) before a final extension stage (72 °C, 7 min). The PCR products were purified using the AMPure magnetic bead cleanup protocol (Agencourt Bioscience Corp, Beverly, MA, USA). Purified amplicons were then sequenced by Sanger sequencing using dye-labeled terminators. Sequence determinations from Sanger reaction products were generated on ABI 3730 analyzers (Applied Biosystems, Carslbad, CA, USA) at the Genomics BioSci &Tech (Taipei) and at the Center of Biotechnology (National Taiwan University). *COI* and *12S* genes were sequenced only from 3′ ends due to the relatively short length (<750 bp), while *RH*, *RAG1*, *ZIC1*, *ENC1* genes were sequenced from both 5′ and 3′ ends (forward and reverse sequencing).

The obtained DNA chromatograms were edited and assembled using CodonCode Aligner 7.1.2 (CodonCode Corporation, Dedham, MA, USA). Some problematic bases with low qualities were checked manually by eyes and discarded if they were unable to make the base calls. In addition, the nucleotide sites in nuclear gene sequences appearing to be heterozygote were recorded into degenerate base symbols (W for A or T; S for G or C; M for A or C; K for G or T; R for A or G; and Y for C or T). The edited sequences of each protein-coding gene (*COI*, *RH*, *RAG1*, *ZIC1*, and *ENC1*) were organized and aligned by eyes based on the inferred amino acid translation with Sequence Alignment Editor (Se-Al) v2.0a11 (Rambaut, 2002). For the *12S* gene sequences, a preliminary alignment was conducted using the automatic multiple-alignment program MUSCLE (Edgar, 2004), then adjusted manually by eye with Se-Al.

### Datasets and analytical methods

#### Datasets

In addition to the sequences newly obtained through laboratory work, we also analyzed sequences from three nuclear genes (*RAG1*, *ZIC1*, *ENC1*) and one mitochondrial gene (*COI*) for 15 aulopiform taxa, as published by Davis (2010) and available in GenBank. Additionally, we incorporated published sequences from diverse sources (Fig. S2). Based on these sequences, we compiled four different datasets for the analyses involved this study: (1) the combined nuclear gene dataset (NC); (2) the mitochondrial genome dataset, which includes only protein-coding gene sequences (mitogenome); (3) the mitochondrial *COI* gene dataset (*COI*), and (4) the combined *12S* and *COI* gene dataset (*12S* +*COI*). The first two datasets (NC and mitogenome) were used to infer the high-level phylogeny of the Aulopiformes, with one outgroup taxon from the Ateleopodidae included, while the *COI* and *12S+COI* datasets were utilized to investigate intra-familial phylogenetic relationships within the Synodontidae and to evaluate species-level hypotheses. Phylogenetic analyses based on nuclear and mitochondrial genome data revealed that the Synodontidae, as traditionally defined, is polyphyletic, comprising two independent lineages corresponding to the two recognized subfamilies. Consequently, each of the *COI* and *12S* +*COI* datasets was further subdivided into two sub-datasets, corresponding to these lineages, for subsequent analyses. Finally, to optimize the datasets, problematic sequences that were either too short or misidentified, or showed signs of contamination (*i.e.,* sequences belonging to non-synodontid fishes, as confirmed by BLAST search or by the results of preliminary phylogenetic analysis), were removed from the analyses.

#### Phylogenetic reconstruction and divergence time estimation

To infer the phylogeny, a partitioned maximum-likelihood (ML) method as implemented in the program RAxML version 8.2.12 12 (Stamatakis, 2014) was carried out with GTR+G nucleotide substitution model. For the NC dataset and mitogenome dataset, 12 and 39 partitions were assigned with respect to codon positions of each gene (four and 13 genes), respectively. For the *COI* datasets, three partitions were set with respect to codon positions of the gene. For the *COI+12S* datasets, four partitions were set with respect to gene and codon positions of the gene. In each analysis, nodal support was assessed by bootstrapping (Felsenstein, 1985) with the ML criterion based on 1,000 pseudo-replicates, except for the *COI* datasets, which were analyzed with 100 pseudo-replicates due to the large data size. The phylogenetic trees were viewed and edited with FigTree v1.4.4 (Rambaut, 2012).

To estimate the divergence times of aulopiform lineages, the NC dataset was used to reconstruct a fossil-calibrated time tree under Bayesian criteria using BEAST v.2.6.7 (Drummond & Rambaut, 2007). The analysis employed a relaxed lognormal clock and the Yule model (Drummond et al., 2006). 12 partitions were assigned with respect to gene and codon positions. The best-fit model for each partition was determined using ModelTest-NG (Darriba et al., 2020) (Table S3). Trees were linked across partitions, whereas clocks were set unlinked.

The phylogenetic tree was time-calibrated using two aulopiform fossil records. The first fossil calibration was based on †*Nematonotus* spp. This fossil genus was initially noted for its osteological similarity to *Aulopus* (Rosen & Patterson, 1969) and was subsequently assigned to the Aulopidae by Rosen (1973). However, the Aulopidae is paraphyletic with respect to Harpadontinae in the phylogenetic analysis based on NC dataset (see result); the calibration point was thus set at the most recent common ancestor (MRCA) of Aulopidae and Harpadontinae using a hard minimum and soft maximum corresponding to the stratigraphic age of this earliest known aulopid fossil (93–96 Ma). The second calibration point was based on †*Atolvorator longipectoralis*, which is the oldest known aulopiform fossil from the Barremian stage of the early Cretaceous (125–130 Ma) (Gallo & Coelho, 2008). This fossil was hypothesized to be closely related to the crown aulopiform lineage of alepisauroids (Kriwet, 2003). In the nuclear gene-based phylogenetic analysis (see result), families traditionally assigned to Alepisauroidei form a distinct group with several other aulopiform families, excluding Aulopidae and Synodontidae. Due to the limited resolution within this group, the calibration point was conservatively applied to the MRCA of all the included families. This placement reflects the minimum age of the group to which the fossil most likely belongs, and avoids overestimating the crown age of Aulopiformes. The two calibration points were implemented using an exponential distribution for divergence time estimation.

Additionally, to avoid inflated or undefined ages and to improve overall reliability of the time estimates, we contained the root node, representing the origin of Neoteleostei, using an exponential prior with an offset at 155 Ma, corresponding to the mean age proposed by Davis & Fielitz (2010) (mean 155 Ma; 95% HPD = 139–176 Ma)*.* For this calibration we used an effective calibration range of 155–170 Ma, a conservative approach that remains well within the HPD in Davis & Fielitz (2010). The age of this secondary calibration is mostly overlapped with most recent divergence-time estimates for major clades of ray-finned fishes (Near et al., 2012; Hughes et al., 2018) and is therefore considered reliable.

For each analysis, four independent runs of 100 million MCMC generations were performed and sampled every 10,000 generations. Each run was initiated from a random starting time tree. The resulting log files and trees files from the four independent runs were removed 15% as burn-in and combined using LogCombiner v.1.7.5. The combined log files were checked with Tracer v.1.7.2 (Rambaut, Drummond & Suchard, 2007) to ensure that all parameters had effective sample sizes (ESS) exceeding 200. The maximum clade credibility (MCC) tree with mean divergence times were generated from the combined tree samples from TreeAnnotator v.2.6.7 (Rambaut & Drummond, 2012).

#### Species delimitation

DNA barcoding has proven to be a useful tool for species identification, offering the advantages of short fragment lengths and conserved regions at the intraspecific level (Ivanova et al., 2007; Ward et al., 2008). In this study, the *COI* gene marker was selected for molecular analyses aimed at delimiting species using two distinct programs: the Assemble Species by Automatic Partitioning (ASAP) (Puillandre, Brouillet & Achaz, 2021) and the Bayesian Poisson Tree Processes (bPTP) model (Zhang et al., 2013). Both approaches cluster nucleotide sequences into operational taxonomic units (OTUs) or putative species without a priori species hypothesis. The ASAP was performed at the web interface under the *K2P* distance, and the bPTP was conducted at the web interface (https://species.h-its.org/) with 500,000 Markov-Chain Monte Carlo (MCMC) generations, the default parameter setting, and an input tree reconstructed using ML method with RAxML. Congruent results between ASAP and PTP were considered as potentially supporting the inferred species. In addition, we used corrected pairwise nucleotide genetic distances (*K2P*) of the *COI* gene, together with morphological data (when voucher specimens were available), to assess the results of ASAP and bPTP. A 3% *COI* divergence threshold was preliminarily applied to evaluate species boundaries among closely related taxa, following Ward (2009). Morphological examinations were based on commonly used diagnostic traits, including head and eye proportions, interorbital space, fin lengths, body and fin coloration and markings, as well as meristic counts, for lizardfishes.

Moreover, we incorporated geographical distribution data to evaluate the potential reproductive isolation among delimited species. Following the approach of Kekkonen & Hebert (2014), only sympatric sister clusters were treated as distinct lineages, whereas allopatric taxa were merged when no other strong evidence (significant genetic and/or morphological divergence) was shown. The determination of the sympatry of the inferred sister OTUs is based on previously described geographical regions, such as the Greater Caribbean (GC), Brazilian provinces (BP), tropical East Atlantic (TEA), East China Sea (ECS), South China Sea (SCS), Sahul Shelf and Australia (SAS), Central West Pacific (Papua New Guinea + New Caledonia) (CWP), and Indian Ocean (IO) for benthic species living in shallow to mid waters of continental shelfs (Luiz et al., 2012; Hung, Russell & Chen, 2017; Lo et al., 2017).

## Results

### Phylogenetic inferences

#### Combined nuclear gene tree

**Figure 3:**
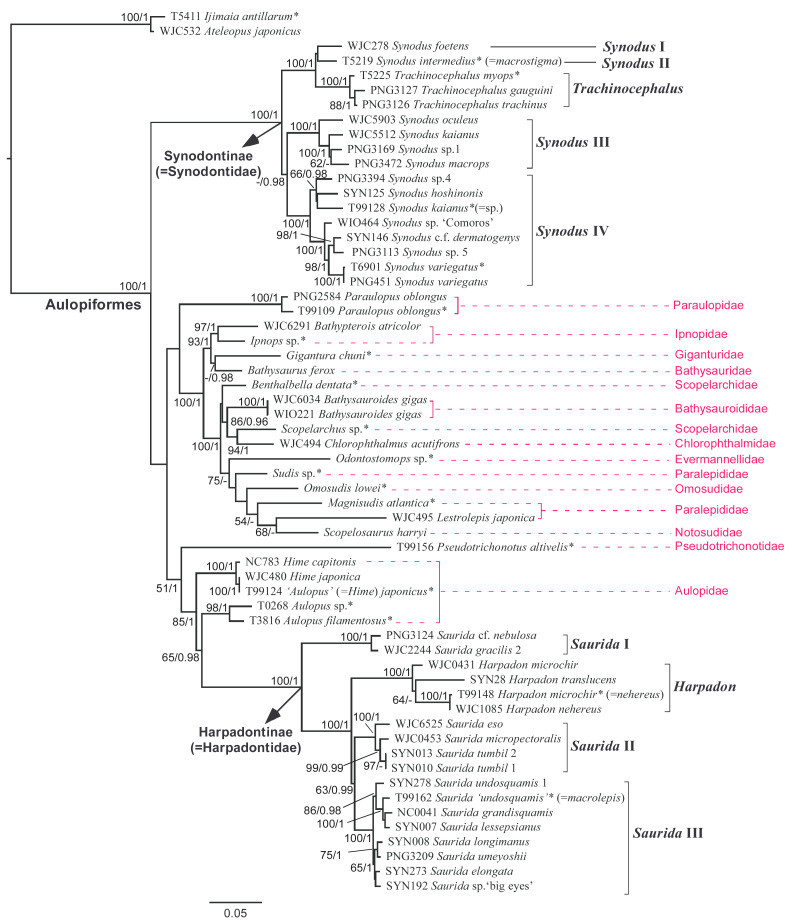
Phylogenetic tree of synodontids and their aulopiform allies reconstructed using partitioned maximum-likelihood (ML) method based on the combined nuclear gene dataset (NC). Branch lengths are proportional to inferred nucleotide substitutions. Numbers at the nodes indicate ML bootstrap values (BP)/Bayesian posterior probabilities (PP) (inferred using BEAST v.2.6.7), with values below 50 (BP) or 0.95 (PP) not shown. Sequences obtained from a previous study (Davis, 2010) are marked with an asterisk (*) after the species name. Corrected species names for sequences misidentified in Davis (2010) are provided in parentheses following the taxon name.

The NC dataset included newly generated sequences of four nuclear genes (*RH*, *RAG1*, *ZIC1*, and *ENC1*) obtained from 38 individuals compiled with three nuclear genes (*RAG1*, *ZIC1*, *ENC1*) from 22 taxa published by Davis (2010) (Table S4). In total, the dataset represented 60 taxa, including 35 taxa from the family Synodontidae, 23 taxa from other aulopiform families, and two outgroups from the family Ateleopodidae (Ateleopodiformes). All of the four synodontid genera were included in the analysis, with 14 taxa from *Synodus*, three from *Trachinocephalus*, 14 from *Saurida*, and four from *Harpadon* (Fig. 3; Tables S1; S4). The other aulopiform representatives included 13 out of 15 other families namely Aulopidae, Bathysauridae, Bathysauroididae, Chlorophthalmidae, Evermannellidae, Giganturidae, Ipnopidae, Notosudidae, Omosudidae, Paralepididae, Paraulopidae, Pseudotrichonotidae, and Scopelarchidae. The final trimmed matrix comprised 3,966 aligned base-pairs and none of stop codon were found along the gene sequences.

From the result of partitioned ML analysis, the families Aulopidae, Paralepididae, Scopelarchidae, and Synodontidae were not resolved as a monophyletic group. Although the two subfamilies of Synodontidae (Synodontinae and Harpadontinae) each formed highly supported clades, they were not sister to each other (Fig. 3). The Synodontinae appeared to be the first-evolved lineage of the Aulopiformes although this relationship was not supported. In contrast, the Harpadontinae was nested within the Aulopiformes, sister to *Aulopus*. These two together grouped with another aulopid genus *Hime* and formed a well-supported clade. However, the other previously proposed “synodontid” allies such as taxa from Paraulopidae and Pseudotrichonotidae did not cluster together with them. Finally, a clade comprising the remaining ten sampled aulopiform families was resolved with the highest support value (Fig. 3).

At the genus level, two of the four currently recognized synodontid genera (*Trachinocephalus* and *Harpadon*) were resolved as monophyletic groups with highest support value, while the other two (*Synodus* and *Saurida*) did not (Fig. 3). Within the “*Synodus”* and “*Saurida*”, four and three main lineages can be defined according to high bootstrap support values (BP) and high Bayesian posterior probabilities (PP) (Fig. 3). The first and second *Synodus* lineages, represented by *S. foetens* (Linnaeus, 1766) and *S. intermedius* (Spix & Agassiz, 1829), respectively, formed a strongly supported clade with *Trachinocephalus*. Notably, these two lineages are all common in the western Atlantic. The third and fourth lineages comprised common species in the IWP region including *S. oculeus* Cressey, 1981 (Cressey, 1981), *S. macrops* Tanaka, 1917, *S. kaianus* (Günther, 1880), *S. hoshinonis* Tanaka, 1917, *S. dermatogenys* Fowler, 1912, *S. variegatus* plus a few potential unknown species (Fig. 3). The polyphyletic *Saurida* consisted of three lineages. However, the distribution of these lineages only weakly accorded with geographical region, with most of the species occurring in the IWP region. The first lineage included *Saurida* sp. (cf. *nebulosa*) and *S. gracilis*, and was sister to the remaining lineages of Harpadontinae, with the highest support value. The second lineage included *S. eso*, *S. tumbil* (Bloch, 1795), and *S. micropectoralis* Shindo & Yamada, 1972. The third lineage comprised *S. undosquamis*, *S. macrolepis* Tanaka, 1917, *S. grandisquamis* Günther, 1864, *S. lessepsianus* Russell, Golani & Tikochinski, 2015*, S. longimanus* Norman, 1939, *S. umeyoshii*, *S. elongata* and an unknown species found in Thailand and Taiwan. However, the inter-relationships among *Harpadon*, *Saurida* I and II remain unresolved (Fig. 3).

#### Mitogenome tree

The mitogenome dataset included sequences data of 13 protein-coding genes of 43 mitochondrial genomes available from the Genbank. This dataset involves 25 taxa from the family Synodontidae, 17 taxa from other aulopiform families, and one outgroup from the family Ateleopodidae. All of the four synodontid genera were included in the phylogenetic analysis (Table S4). The other aulopiform representatives included 13 out of 15 other families: Aulopidae, Alepisauridae, Anotopteridae Bathysauridae, Chlorophthalmidae, Evermannellidae, Giganturidae, Ipnopidae, Notosudidae, Omosudidae, Paralepididae, Pseudotrichonotidae, and Scopelarchidae. The final trimmed matrix comprised 11,424 aligned base-pairs and none of stop codon were found along the gene sequences. However, during the alignment process, two *Harpadon nehereus cytb* sequences retrieved from GenBank required the insertion of five gaps at position 874 to properly align with other taxa. This suggests that there might be some issues with these sequences (MH204885 and JX534239) in the GenBank database.

**Figure 4:**
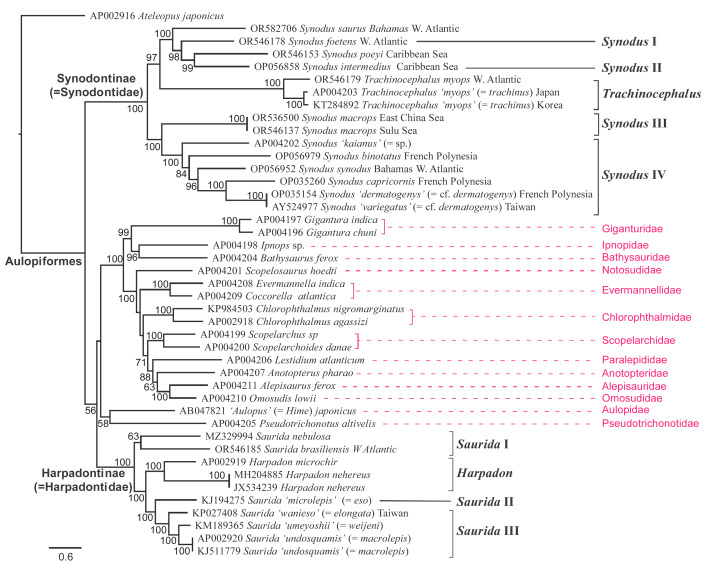
Phylogenetic tree of synodontids and their aulopiform allies reconstructed using partitioned maximum-likelihood (ML) method based on the mitochondrial genome (mitogenome) dataset. Branch lengths are proportional to inferred nucleotide substitutions. Numbers at the nodes indicate ML bootstrap values (BP), with values below 50 not shown. Corrected species names for misidentified GenBank sequences are provided in parentheses following the taxon name.

Figure 4 illustrates the mitochondrial phylogenomic tree inferred through partitioned maximum-likelihood analysis. The topology of this tree is comparable to that of the combined nuclear gene tree, despite some differences in taxonomic coverage between the datasets. Both analyses consistently indicated that the family Synodontidae is not monophyletic, although its two subfamilies exhibit strong support for monophyly. Within each subfamily, the main lineages identified in the combined nuclear gene analysis were also evident in the mitogenome analysis. Additionally, we confirmed the close evolutionary affinity of the western Atlantic *Synodus* I and II with two other taxa, *S. saurus* and *S. poeyi*, which co-occur in the same geographic region. Compared to the combined nuclear gene results, the mitogenome analysis provided stronger support for the interrelationships among the major synodontid clades or lineages.

On the other hand, the hypothesized close relatives of Synodontidae—Aulopidae and Pseudotrichonotidae—did not cluster with either synodontid subfamily. These two taxa appeared to be the sister group of a well-supported clade comprising 11 other sampled aulopiform families, including Alepisauridae, Anotopteridae, Bathysauridae, Chlorophthalmidae, Evermannellidae, Giganturidae, Ipnopidae, Notosudidae, Omosudidae, Paralepididae, and Scopelarchidae. Within this clade, a few well-supported sister-group relationships were inferred, most notably among the following pairs: Bathysauridae/Ipnopidae, Giganturidae/(Bathysauridae+Ipnopidae), and Alepisauridae/Omosudidae (Fig. 4).

#### COI gene tree

**Figure 5:**
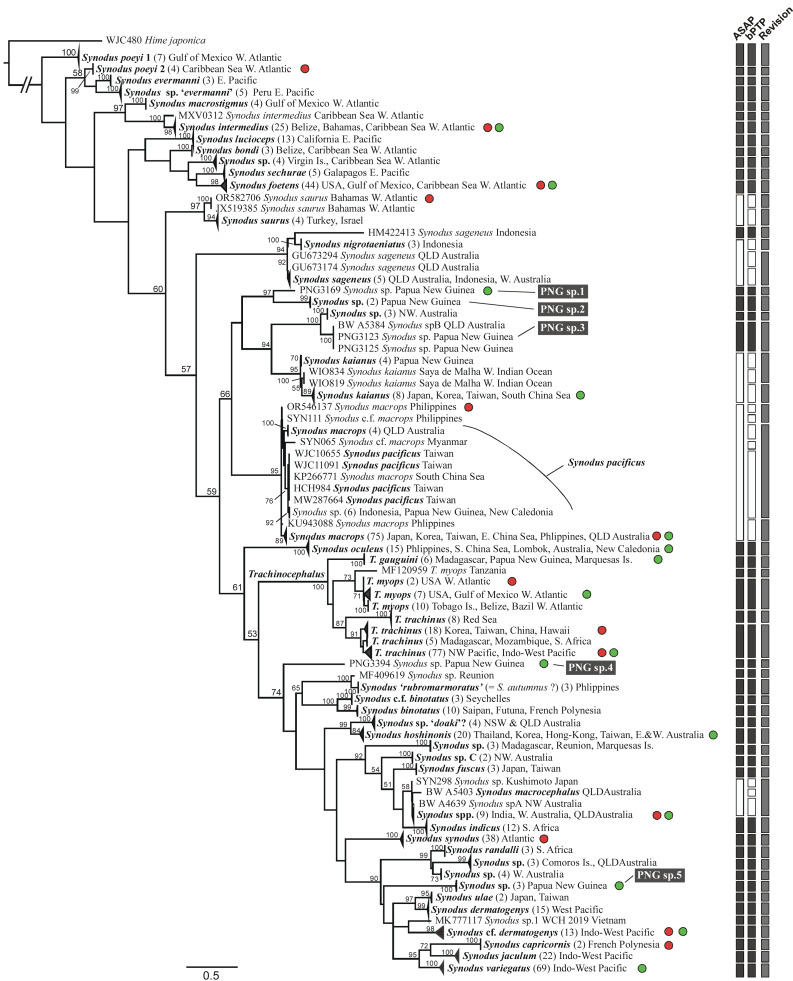
*COI* gene tree of Synodontinae (=Synodontidae, newly defined in this study) inferred using the maximum-likelihood (ML) method, alongside species delimitation analyses (ASAP and bPTP; vertical bars on the right). Branch lengths are proportional to inferred nucleotide substitutions. Numbers at the nodes indicate ML bootstrap values (BP), with values below 50 (BP) not shown. Red circles denote taxa included in the higher-level mitogenome phylogenetic analysis, while green circles highlight selected representative taxa from the main resolved *COI* lineages for the combined *COI* and *12S* gene analysis. Numbers within the parentheses shown after the taxon names indicate the number of sequences within each collapsed clade or lineage. Congruent species delimitation results across both methods are indicated by black-colored bars.

**Figure 6:**
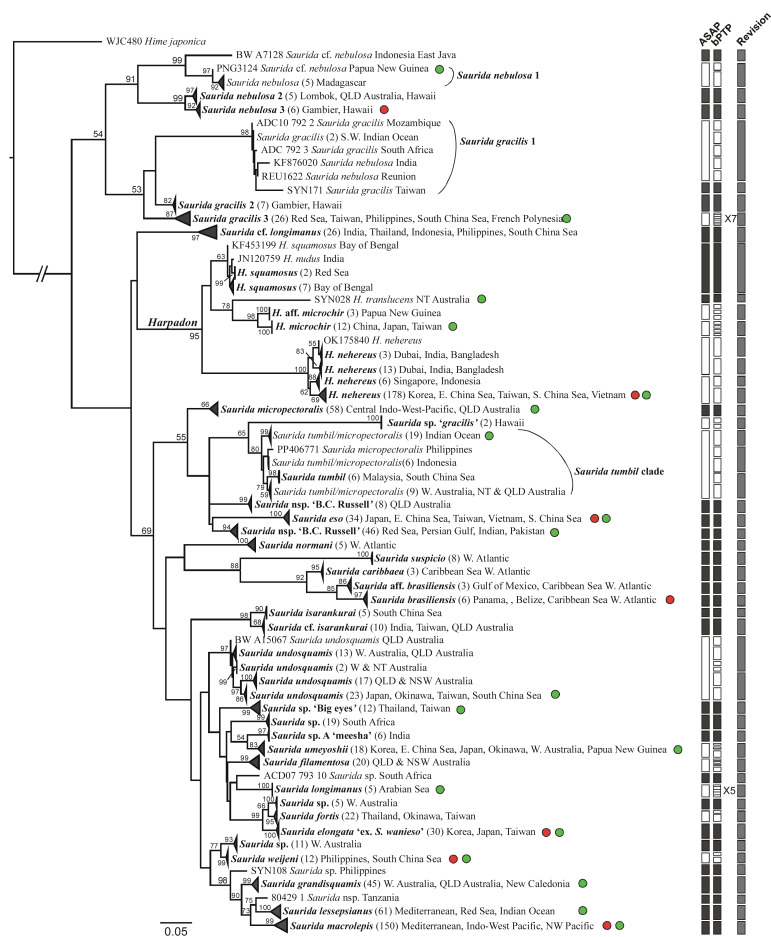
*COI* gene tree of Harpadontinae (=Harpadontidae, newly defined in this study) inferred using the maximum-likelihood (ML) method, alongside species delimitation analyses (ASAP and bPTP; vertical bars on the right). Branch lengths are proportional to inferred nucleotide substitutions. Numbers at the nodes indicate ML bootstrap values (BP), with values below 50 (BP) not shown. Red circles denote taxa included in the higher-level mitogenome phylogenetic analysis, while green circles highlight selected representative taxa from the main resolved *COI* lineages for the combined *12S* and *COI* gene analysis. Numbers within the parentheses shown after the taxon names indicate the number of sequences within each collapsed clade or lineage. Congruent species delimitation results across both methods are indicated by black-colored bars.

The high-level phylogenetic analyses confirmed the two subfamilies were two independent monophyletic groups. Therefore, the compiled synodontid *COI* gene sequences were separated into two datasets with respect to each subfamily (Synodontinae: *Synodus* + *Trachinocephalus*; Harpadontinae: *Harpadon* + *Saurida*). Figures 5 and 6 present the phylogenetic trees of the two subfamilies based on these two separated datasets, with sequences from some monophyletic groups collapsed. The datasets comprised 1,017 sequences (70 newly obtained) and 673 sequences (165 newly obtained), respectively, with 615 aligned base-pairs for each. Detailed information on the *COI* trees, including the full names of sequences, GenBank accession numbers, and their associated sample IDs, is provided in the Supplementary Data (Figs. S3–S6). Despite the low bootstrap supports, the relationships among the resolved major lineages in both *COI* gene trees were consistent with the results of the combined nuclear gene tree and mitogenome tree (Figs. 3–4). Within each genus, most of the nodes that indicated species level were supported by high bootstrap value. Therefore, the trees revealed the grouping of recognized species. However, many doubtful clades were observed in the sequences retrieved from other sources, with two or more ‘species’ names showing in one monophyletic group (Figs. 5–6; Figs. S3–S6). This was likely due to high levels of misidentification, especially for sources such as GenBank and BOLD (Appleyard et al., 2025). To infer the “true” species name of each monophyletic clade, the identity of the species was reconfirmed by examining the deposited specimens in NTUM, NMMBA, and NTM collections or validated by the clade that included samples collected from type locality.

Within the subfamily Synodontinae, the genus *Trachinocephalus* was clearly a monophyletic group, with the highest bootstrap support value (Fig. 5). However, the results showed the presence of at least five lineages for this genus. The known widespread species *T. myops* comprised two genetically and geographically distinct lineages (*T. ‘myops’* from western Atlantic and *T. ‘myops’* from Tanzania). The IWP distributed *T. trachinus* was also found to contain two geographically distinct lineages: one from the Red Sea and the other from the broader IWP region (Fig. 5). The genus *Synodus* is paraphyletic with respect to the monophyletic *Trachinocephalus*. Within the genus, 54 well-supported clades or lineages (bootstrap value ≥ 80) were identified at or above the species level (Fig. 5). The offshore lizardfish, *Synodus poeyi* Jordan, 1887, was found to be polyphyletic, comprising two distinct lineages: one in the Caribbean Sea and the other in the Gulf of Mexico in the western Atlantic. The commonly encountered reef-associated species, *S. dermatogenys*, contains independently evolved cryptic lineages, with one restricted to the West Pacific and the other distributed widely across the IWP region (Fig. 5). For another reef-associated species, *S. variegatus*, sequences associated with this name appear in three distinct clades of *Synodus* species, including the *S. cf. dermatogenys* clade. Nonetheless, we propose that the clade comprising the majority of *S. variegatus* specimens, with sample localities spanning the IWP region and including the type locality (Central West Pacific), likely represents the ‘true’ *S. variegatus*. In contrast, those found exclusively in western Australia may represent a potential new species (Fig. 5; Fig. S3). Further detailed taxonomic investigation of this species is warranted.

In the subfamily Harpadontinae, the genus *Harpadon* formed a well-supported monophyletic group (BP = 95; Fig. 6). The analysis included five recognized species from this genus, revealing three main clades (BP ≥ 62). The first clade contained two morphospecies, *H. nudus* Ganga & Thomas, 2016 and *H. squamosus* Alcock, 1891, while the second comprised *H. translucens* Saville-Kent, 1889 and *H. microchir* Günther, 1878. The third clade consisted of a single species, *H. nehereus* (Hamilton, 1822). The morphospecies *H. microchir and H. nehereus* can each be further divided into at least two distinct subclades.

The genus *Saurida* was found to be polyphyletic. The *COI* tree identified 49 well-supported clades or lineages (BP ≥ 80) at or above the species level. While most morphospecies were monophyletic, some exceptions were observed. Notably, *S. nebulosa* (Valenciennes, 1850), *S. gracilis*, and *S. longimanus* each consisted of multiple independently evolved lineages that were genetically and geographically distinct, highlighting the need for further detailed taxonomic investigation (Fig. 6). In addition, we observed that the species previously recognized as part of the *S. undosquamis* complex did not form a monophyletic group.

#### Combined 12S and COI gene tree

**Figure 7:**
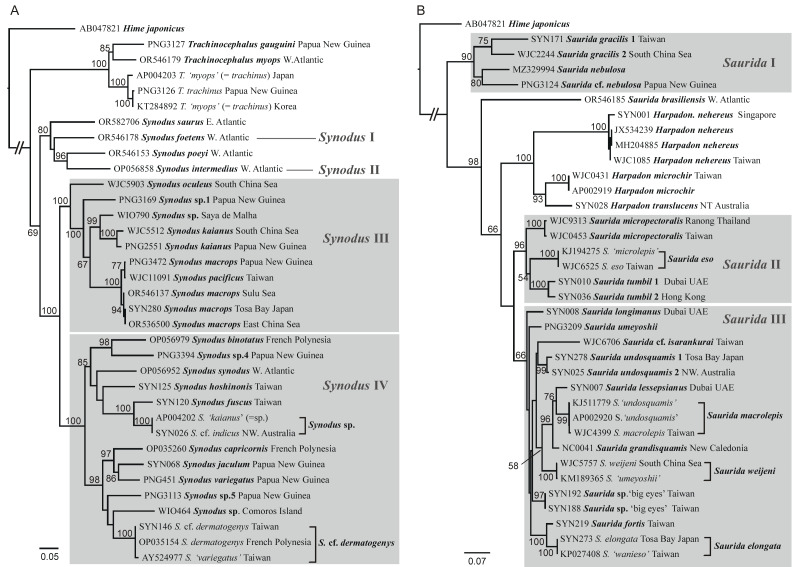
Combined *12S* and *COI* gene trees of Synodontidae (A) and Harpadontidae (B) inferred using the maximum-likelihood (ML) method. Branch lengths are proportional to inferred nucleotide substitutions. Numbers at the nodes represent ML bootstrap values (BP), with values below 50 (BP) omitted. Gray rectangle boxes highlight the main lineages/clades resolved in the ML analyses of the NC and mitogenome datasets.

Figure 7 illustrates the phylogenetic trees of the two subfamilies, reconstructed from the combined *12S* and *COI* gene datasets, including representative taxa from each of the main clades/lineages resolved in the *COI* trees. The datasets contained 2,586 and 2,583 aligned base-pairs, respectively. The inclusion of the more slowly evolving *12S* gene marker significantly increased bootstrap support for deeper nodes compared to the analysis based solely on *COI* sequences. The inferred phylogenetic relationships were also more aligned with those derived from NC and mitogenome datasets. Notably, the monophyletic groups *Trachinocephalus* and *Harpadon*, along with the distinct major clades of *Synodus* and *Saurida*, were all identified in the combined *12S* and *COI* gene analyses, with high support values (except *Saurida* III; BP = 66 only). In addition, these results also revealed potential instances where species names associated with published mitogenome data were incorrectly assigned, likely due to previously unsolved issues in taxonomy or misidentification. For example, *Trachinocephalus ‘myops’* found in Japan and Korea (GenBank accession nos: AP004203 and KT284892) should be assigned to the IWP distributed species *T. trachinus* (Wang et al., 2018), while *Saurida ‘microlepis’* (GenBank accession no: KJ194275) and *S. ‘undosquamis’* (GenBank accession nos: KJ511779 and AP002920), which were often confused with other *Saurida* species previously, should be recognized as *S. eso* and *S. macrolepis*, respectively, according to updated taxonomic information (Russell, Motomura & Furuhashi, 2025). Finally, despite being morphologically distinct, we observed that *Synodus pacificus* Ho, Chen & Shao, 2016 was nested within *S. macrops*, warranting further detailed investigation.

### Divergence time estimation

**Figure 8:**
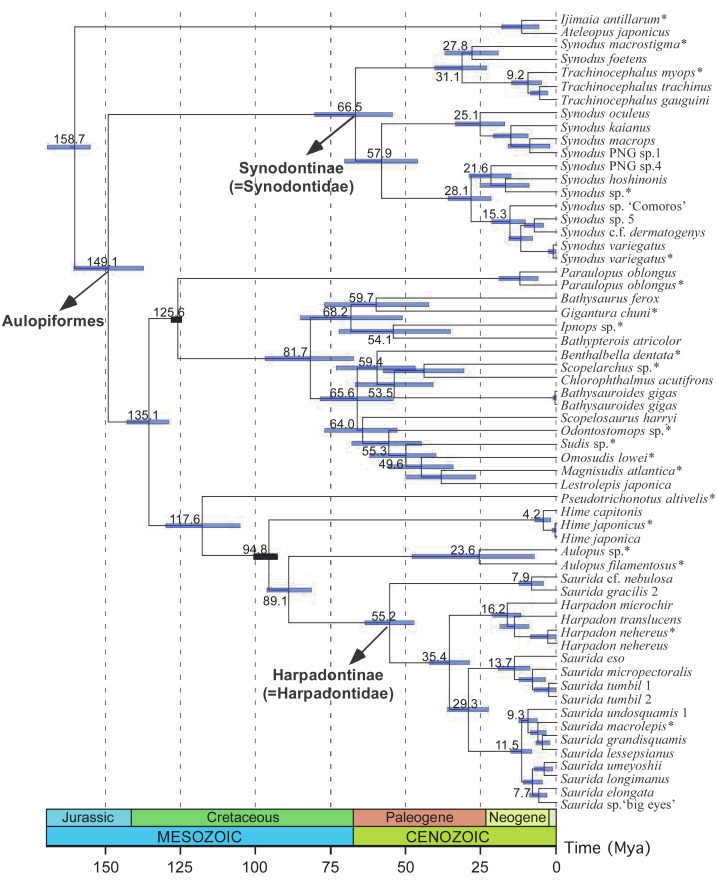
Fossil-calibrated time tree of synodontids and their aulopiform allies inferred from NC datasets. The scale is in millions of years. Numbers at the nodes indicate the mean clade ages, with blue bars representing the 95% highest posterior density (HPD) intervals. Fossil-calibrated points are shown as black bars. Sequences obtained from a previous study (Davis, 2010) are marked with an asterisk (*) following the species name.

From the inferred time tree based on Bayesian divergence time analysis, the reconstructed phylogeny was almost identical to the topology of the combined nuclear gene tree under ML criteria except a few nodes with weak statistic support (*i.e.,* within the clade comprising taxa from *Odontostomops*, *Sudis*, *Omosudis*, *Magnisudis*, *Lestrolepis*, and *Scopelosaurus*), thus reconfirming the phylogenetic relationships of the family, especially the monophyly of the two subfamilies, Synodontinae and Harpadontinae (Fig. 3). The origin of the Synodontinae clade was estimated to be at 66.5 Ma (95% HPD: 54.5–80.1), while the Harpadontinae clade was estimated to have a more recent occurrence at around 55.2 Ma (95% HPD: 47.4–63.5). Most of the aulopiform families suddenly appeared around from 50 to 68 Ma (Fig. 8).

### Species delimitation

Two analytic programs were used for the species delimitation among the collected *COI* sequences from the Synodontidae (two datasets for two subfamilies). For the subfamily Synodontinae, the ASAP analysis was conducted by partitioning all the individuals into 50 OTUs (or putative species). However, 63 entities were clustered through the bPTP analysis, which was based on a maximum likelihood partition. For the subfamily Harpadontinae, ASAP analysis was conducted by partitioning all the individuals into 47 OTUs, while bPTP analysis indicated much higher number of 85 OTUs with a maximum likelihood partition. The conflict between the results of the two analyses are discussed and addressed in the discussion section. In total, 56 synodontine and 51 harpadontine species were delimited based on multiple lines of evidence in this study (Figs. 5 and 6).

## Discussion

### High-level phylogeny

#### Phylogenetic position(s) of the synodontidae within the aulopiformes

Currently, the monophyly of Aulopiformes is supported by nine morphological synapomorphies (Baldwin & Johnson, 1996; Johnson, 1992; Sato & Nakabo, 2002; Davis, 2010). Davis (2010) further demonstrated the monophyly of the Aulopiformes based on a combination of molecular and morphologic evidence with thorough sampling of representatives from major euteleost lineages and extensive aulopiform families. However, the phylogenetic position and taxonomic status of the synodontids have long been controversial within the Aulopiformes (Gosline, Marshall & Mead, 1966; Rosen, 1973; Rosen, 1985; Sulak, 1977; Johnson, 1982; Baldwin & Johnson, 1996; Sato & Nakabo, 2002; Davis, 2010; Chaiyapo, 2013) (Fig. S1). Within the order, the family Synodontidae has the closest relationship with the Aulopidae and Pseudotrichonotidae as supported by shared synapomorphies such as the elongated and widely separated posterior pelvic processes (Baldwin & Johnson, 1996). The two subfamilies of the Synodontidae were previously assigned into one group based on the structural characters such as a strong premaxilla dominated upper jaw; reduced or absence of supramaxillae; a modified gill raker with cluster of short gill teeth; a pointed snout; a high number of branchiostegal rays (12–26), and a dioecious mode of reproduction (Sulak, 1977). However, some of these synapomorphies are found also in other aulopiform species. For example, the strong premaxilla can also be observed in the genus *Bathysaurus*, and dioecious reproduction occurs in *Pseudochronotus*, *Aulopus* and *Paraulopus* (Sulak, 1977; Davis, 2010).

From the high-level phylogenetic analyses conducted in this study (Figs. 3–4), the family Synodontidae was not resolved as a monophyletic group that corresponds to previous classifications proposed by Gosline, Marshall & Mead (1966), Rosen (1973), and Johnson (1982). In addition, the multi-nuclear gene result showed that Harpadontinae has a closer relationship to the family Aulopidae, a result also observed by Sulak (1977). Considering that the classification of species should reflect their actual evolutionary relationships, the placement of the two subfamilies under the same family Synodontidae might no longer be appropriate. Based on molecular and morphological data, herein we propose elevation of the two subfamilies into two independent families within the Aulopiformes, Synodontidae and Harpadontidae sensu Rosen (1973), as follows:

Family Synodontidae Gill, 1861: 53, type genus *Synodus*

**Table:** 

Genus *Synodus* Scopoli, 1777 (Scopoli, 1777): 449
Genus *Trachinocephalus* Gill, 1861: 53

Family Harpadontidae Bleeker, 1875, type genus *Harpadon*

**Table:** 

Genus *Harpadon* (subgenus of *Saurus*) Lesueur, 1825: 50
Genus *Saurida* Valenciennes in Cuvier & Valenciennes, 1850: 499

This classification will be followed in the rest of the discussion. To distinguish the two newly proposed families, despite the common characters, there are still some apparent morphological variations that can be observed. The most obvious dissimilarity is the number and relative length of the pelvic fin rays (species within the Synodontidae have eight pelvic fin rays, with the inner one much longer than the outer; while species within the Harpadontidae have nine pelvic fin rays of almost equal length (Fig. 1). Other morphological differences can also be found, such as the presence of toothed vomer and supramaxillae in Harpadontidae but its absence in Synodontidae. According to Chaiyapo (2013), both families are unambiguously supported by several synapomorphic characters: the family Synodontidae is supported by 16 synapomorphic characters, such as the rostral cartilage enlarged and loosely attached with the ethmoid, and the lowermost actinost expanded; while species within the Harpadontidae share 11 synapomorphies, including teeth on the ectopterygoid, and the presence of a gap between the fourth basibranchial and fifth ceratobranchial (Chaiyapo, 2013). This morphological differentiation of the two groups further supports the validation of two independent lizardfish families proposed according to the result of the high-level phylogeny analyses in this study.

Another additional finding in this study is that the family Aulopidae is also paraphyletic. At least two independent lineages exist in this family, *Hime* and *Aulopus*. *Hime* was first published to describe the Pacific aulopids and was often regarded as a junior synonym of *Aulopus* (Starks, 1924; Sulak, 1977; Thompson, 1998; Thompson, 2002; Prokofiev, 2008; Nelson, Grande & Wilson, 2016). Although the genus *Hime* was resurrected by Parin & Kotlyar (1989), subsequent studies found no evidence in either morphology nor genetics that support the validity of this genus, and synonymized *Hime* in the genus *Aulopus* (Baldwin & Johnson, 1996; Davis, 2010). However, with enhanced molecular markers, our high-level phylogeny found a distinct pattern of genetic variation between these two genera (Fig. 3), which support *Hime* as a valid genus of the Aulopidae (Randall, 2009; Fricke et al., 2014; Gomon, Struthers & Stewart, 2013; Gomon & Struthers, 2015). Further systematic investigations by including more aulopid taxa, notably, the species from the remaining genera, *Latropiscis* and *Leptaulopus*, are needed.

#### Phylogeny of the lizardfishes

In this study, a reliable phylogeny of lizardfishes belonging to the two newly defined families was inferred through the “top-down” and “bottom-up” processes. The bottom-up process began with a preliminary phylogenetic analysis of collected *COI* sequences. This step facilitated the grouping of numerous unknown, problematic or misidentified species and provided a basic framework for further understanding the relationships among each major clade. Based on the results of the *COI* trees, 32 and 29 taxa were subsequently selected to represent the major groups of lizardfishes in the top-down process. The top-down approach was then carried out by reconstructing the phylogeny of lizardfishes using combined datasets of *12S* and *COI* sequences, as well as the high-level phylogeny of Aulopiformes using combined nuclear gene sequences. By integrating both approaches, this bidirectional strategy minimized biases arising from misidentifications or uneven sampling of specific groups and provided deeper insights into the evolutionary relationships among lizardfishes.

The phylogeny based on the *COI* gene sequences, however, failed to resolve most intra-generic relationships among lizardfish species. This result may represent a soft polytomy caused by rapid species radiation (Pavlicev & Mayer, 2009), suggesting that the *COI* gene evolves faster than the associated phenotypic traits. Fortunately, this limitation can be addressed by incorporating additional phylogenetic information, such as increasing the number of molecular markers or expanding taxon sampling. Consequently, the phylogenetic inference for lizardfish species discussed here relies primarily on multi-gene trees, while the *COI* gene trees are used to confirm species-level monophyly.

This study represents the first molecular phylogenetic investigation focusing specifically on lizardfishes. Although the dataset does not include all known species in this group due to sampling limitations, the overall phylogeny could still be inferred using the selected representative taxa. The phylogenetic results (Figs. 3–4) reveal that lizardfishes are not monophyletic, contrary to previous taxonomic classifications (Sulak, 1977; Baldwin & Johnson, 1996; Sato & Nakabo, 2002; Nakabo, 2002; Davis, 2010). The broader phylogeny of lizardfishes and their aulopiform relatives reconstructed in this study closely aligns with Davis (2010), which was based on five genetic markers. However, Davis’s phylogeny, derived from a total evidence approach, may have been influenced by morphological characters, leading to the depiction of “Synodontidae” as monophyletic (Fig. 1B).

The inter-generic relationships among lizardfishes were also investigated in this study. The monophyly of *Trachinocephalus* and *Harpadon* was confirmed. However, both *Synodus* and *Saurida* were consistently found to be paraphyletic across all high-level phylogenetic analyses (Figs. 3–4) though *Synodus* appeared to be monophyletic in *12S* plus *COI* gene tree, with weak support (Fig. 7). Regardless of the gene datasets used, both *Synodus* and *Saurida* were divided into at least three major lineages (Figs. 3–4; Fig. 7). This internal division within these genera has not been reported in previous systematic studies (Gosline, Marshall & Mead, 1966; Rosen, 1973; Johnson, 1982; Sulak, 1977; Rao, 1977; Baldwin & Johnson, 1996; Sato & Nakabo, 2002; Davis, 2010).

Species in the genera *Trachinocephalus* and *Harpadon* exhibit highly specialized morphology and habitat adaptations. The genus *Harpadon*, commonly known as “Bombay duck”, is distinguished by its highly specialized features, such as a large gape, an extremely short snout, and elongated jaws, and occupies a pelagic habitat (Sulak, 1977). In the higher-level phylogenetic analyses, *Harpadon* was revealed to be more closely related to *Saurida* II and III, than *Saurida* I, suggesting an evolution of autapomorphic features in *Harpadon.* At the species level, *H. erythraeus* has been reported to inhabit deeper waters compared to its congeners (Klausewitz, 1983). Previous studies have suggested that *Harpadon* species distributed in the IWP region can be divided into inshore and offshore groups based on phenotypic and osteological differences. Offshore species include *H. erythraeus*, *H. squamosus*, and *H. microchir*, while *H. translucens* and *H. nehereus* are categorized as inshore species (Ganga, Thomas & Sukumaran, 2015; Johnson, Langston & Schmitz, 1997). Although this study does not include all recognized species, our phylogenetic analyses strongly support the grouping of *H. translucens* and *H. microchir*, thereby challenging the previously proposed habitat-based classification.

*Trachinocephalus*, commonly known as the blunt-nose lizardfish, represents another specialized group. In our high-level phylogenetic analyses, it was resolved as being more closely related to species in *Synodus* lineages I, II, *S. saurus*, and *S. poeyi* than to other *Synodus* species (Figs. 3–4). Unlike *Synodus*, species in *Trachinocephalus* are characterized by a reduced snout length, giving them a nearly blunt head, and a higher number of anal fin rays (Polanco, Acero & Betancur, 2016). Despite these morphological differences, Chaiyapo (2013) conducted an in-depth examination of osteological and morphological features of several representative lizardfish species and suggested that *Trachinocephalus* shares a most recent common ancestor with *Synodus saurus*, supported by four synapomorphies. While not all aspects of this relationship were resolved, our phylogenetic results—particularly those derived from mitogenome data—are consistent with this hypothesis. Further investigations are still needed to fully clarify the taxonomic status of lizardfishes at the generic level.

### Evolutionary origins and diversifications of the lizardfishes

In terms of the timing of evolutionary origins of lizardfishes, while the divergence times of aulopiform species as a whole have been estimated by Davis & Fielitz (2010), the detailed timeline within the Synodontidae remains unclear due to limited sampling in previous studies. Despite this, the origins of the major aulopiform lineages were dated back to the Early Cretaceous, with most extant families appearing by the Late Cretaceous to the Eocene (Davis & Fielitz, 2010). As most lizardfishes are reef or shelf-associated benthic species, their origins and diversification may have been driven by the progressive recovery of shallow water marine ecosystems after the Cretaceous–Paleogene (K–Pg) mass extinction (∼66 Ma), together with the subsequent expansion of reef habitats between the Paleocene and early Miocene (Veron, 1995; Cowman & Bellwood, 2013; Price et al., 2014; Bellwood, Goatley & Bellwood, 2017; Chen & Borsa, 2020) and the restructuring of trophic networks (Bellwood, Goatley & Bellwood, 2017). Furthermore, lizardfishes are of few families within the order Aulopiformes that exhibit separate sexes—a primitive reproductive characteristic—suggesting an origin earlier than its aulopiform relatives (Davis, 2010). However, these evolutionary inferences cannot be fully validated without a robust time-calibrated phylogeny. The fossil-calibrated time tree reconstructed in this study revealed distinct divergence times for the two lizardfish families (Fig. 8). The divergence time of the newly defined Synodontidae was estimated to have occurred during the end of the Cretaceous (66.5 Ma), while the origin of the newly defined Harpadontidae was dated to the Eocene (55.2 Ma). These estimates align well with the divergence times of Aulopiformes proposed by Davis & Fielitz (2010).

From an evolutionary morphological perspective, lizardfishes share some of the most primitive traits with the stem species of Aulopiformes, including round, laterally directed eyes and a dioecious reproductive strategy (*i.e.,* separate sexes) (Sulak, 1977; Baldwin & Johnson, 1996; Davis & Fielitz, 2010). Sulak (1977) proposed that *Saurida* represents the most primitive member of the previously defined “Synodontidae”, possessing transitional features between *Aulopus* and synodontids, such as an *Aulopus*-like vomer and a long, well-toothed palate characteristic of synodontids. Sulak further hypothesized that the evolutionary order of the four lizardfish genera could be inferred based on the presence or reduction of supramaxillae: two supramaxillae in *Saurida*, one vestigial supramaxilla in *Harpadon*, and none in *Synodus* and *Trachinocephalus*. However, the divergence time estimations and evolutionary relationships inferred in this study do not support Sulak’s assumption. The origin of the Synodontidae is estimated to predate that of the Harpadontidae, and the presence of supramaxillae appears to have evolved independently within these lineages. Furthermore, the presence of a toothed vomer is not unique to Harpadontidae but also occurs in other aulopiform families, such as Aulopidae and Bathysauridae, indicating a closer relationship among these groups (Sulak, 1977). While the origin of the previously defined “Synodontidae” remains contentious, Sulak’s study provides evidence supporting a closer relationship between Harpadontidae and Aulopidae. Since the two families diverged along separate evolutionary lineages at different geological times, shared traits among lizardfishes, such as round eyes and separate sexes, should be interpreted as homoplastic characters resulting from convergent evolution rather than shared ancestry.

### Species level taxonomy and biodiversity

#### Species delimitation

Two programs were applied to help to delimitate species, ASAP and bPTP. As both analytical approaches using single locus data for computation, they can provide a relatively fast and valuable perspective into species boundaries (Kekkonen & Hebert, 2014; Leavitt, Moreau & Lumbsch, 2015). Several disagreements between the results from ASAP and bPTP analysis were observed in this study. This incongruity could be attributed to the difference between the algorithms applied for species delimitation in the two analytical methods. With an input of sequence alignment file, ASAP sorts sequences into hypothetical species (OTUs) based on the detected barcode gap using the recursive approach (Puillandre, Brouillet & Achaz, 2021). The analysis of bPTP needs an input file of an inferred phylogenetic tree, and then the inference of putative species boundaries is made based on a Bayesian implemented Poisson tree processes model. This model considers the number of mutations as speciation or branching events and can infer species boundaries with the phylogenetic species concept (Zhang et al., 2013). However, these two methods were based on single locus data and can only provide a preliminary assessment for species delimitation (Fujisawa & Barraclough, 2013; Leavitt, Moreau & Lumbsch, 2015). The validation of inferred species should also consider other criteria such as genetic diversity, nuclear gene phylogeny, geographic distribution, ecology, and/or morphological difference (Carstens et al., 2013; Kekkonen & Hebert, 2014; Leavitt, Moreau & Lumbsch, 2015; Hung, Russell & Chen, 2017; Lo et al., 2017; Lee et al., 2019; Chen, Lee & Chen, 2024; Hurzaid et al., 2020; Lee et al., 2022). In this study, since many sequences were sourced from public databases without corresponding voucher specimens, the validation of discordant clusters was primarily based on the *COI* gene phylogeny, genetic diversity (estimated using *K2P* distances at the *COI* locus) (Kimura, 1980), geographic distribution data, and, when available, documented morphological features from specimens and taxonomic literature. The following sections detail specific cases of our species delimitation.

Generally, bPTP tended to differentiate sister clusters with geographical differences while ASAP did not. For example, *Synodus saurus* JX519385 and OR582706 (Bahamas) and other *S. saurus* individuals (Israel and Turkey); *Synodus kaianus* (Papua New Guinea), *S. kaianus* (western Indian Ocean) and other *S. kaianus* individuals (Korea, Tosa Bay in Japan, and South China Sea). For *S. saurus*, the formation of strongly supported reciprocal clades and high genetic heterogeneity (5.73%) between the two completely isolated groups (western Atlantic *vs.* Mediterranean Sea) suggests the presence of cryptic species. The genetic divergence estimated using *COI* sequences is significantly higher than the 3% threshold set for the species boundary of sibling marine fishes (Ward, 2009). Therefore, the two OTUs resolved by bPTP is accepted. In contrast, for *S. kaianus*, the genetic differentiation among the three lineages (OTUs) defined by bPTP is less pronounced than for the previous case (ranging from 1.23% to 4.41%). Since, it displays allopatric distributions, the final decision of this cases is based on the approach suggested by Kekkonen & Hebert (2014), by merging them into a single OTU or putative species. This is because the absence of interbreeding between heterospecific organisms can only be assessed in instances of sympatric, but not allopatric, distribution. Similarly, we combined two sister bPTP OTUs of *Saurida* cf. *nebulosa* PNG3124 from Papua New Guinea and five individuals of *S. nebulosa* 1 from Madagascar into a single one (genetic divergence between them: 1.52%). We also merged five bPTP OTUs of *Saurida gracilis* 1 from Taiwan, and several different localities in the Indian Ocean into one, based on the low inter-OTU genetic diversity (ranging from 0.82% to 5.48%) and allopatric distribution. However, it should be noted that this decision is tentative, and further evidence, such as that from morphological examination, is necessary to validate the species delimitation.

In other cases (*Harpadon* aff. *microchir*, *H. microchir*, *Saurida gracilis* 3, *S.* sp. “*gracilis*” Hawaii, *S. umeyoshii*, *S. filamentosa*, *S. longimanus*, *S. fortis*, and *S. weijeni*), while ASAP identified a single OTU, bPTP appeared to overpredict, assigning individuals of the same morphospecies with similar or nearly identical sequences and minimal divergence to separate OTUs. Consequently, we merged these multiple OTUs and accepted the species delimitation results suggested by ASAP.

Finally, some cases are more complex, they necessitate further investigation. For instance, within the *Saurida tumbil* clade, a few sequences of *S. micropectoralis* were included, rendering both morphospecies polyphyletic. Based on the level of genetic diversity, *COI* phylogeny, and the allopatric distribution of sister OTUs, three putative species can tentatively be inferred: *Saurida tumbil* from the Indian Ocean, *S. ‘tumbil’* from the Philippines and Indonesia, and *S. ‘tumbil’* widely distributed from Malaysia and the South China Sea to Australia (Fig. 6). The genetic divergence among these three inferred species ranges from 3.6% to 4.7%. *Since* the type specimens of *S. tumbil* were collected from southeastern India, near Sri Lanka, we suggest that the *Saurida tumbil* from the Indian Ocean may represent the “true” species, while the others could either be *S. micropectoralis* (type locality: Gulf of Thailand, South China Sea), misidentification, or potential new species. Further confirmation through morphological examination of these putative species is underway.

A similar, though not identical, case can be observed in the *Synodus sageneus*, *S. macrops/pacificus*, and *S. macrocephalus/*spp. clades (Fig. 5). In the *S. sageneus* clade, another morphospecies, *S. nigrotaeniatus* Allen, Erdmann & Peristiwady, 2017 is included, making *Synodus sageneus* paraphyletic in the inferred *COI* tree. *S. nigrotaeniatus* is a recently described species based on six specimens collected from Lembeh Strait, North Sulawesi Province, Indonesia. The two species share most morphological and meristic features but differ in markings, with *S. nigrotaeniatus* exhibiting a black midlateral stripe, wider interorbital space, and lower vertebral and lateral-line scale counts (Allen, Erdmann & Peristiwady, 2017). Our genetic analysis shows that *S. nigrotaeniatus* is genetically differentiated from other *S. sageneus* lineages, supporting its species status. Based on these morphological and genetic data along with geographic consideration, we infer that this clade should consist of three separate species, with *S. nigrotaeniatus* being sister to *S. sageneus* (sequence accession no. HM422413), which is also found in Indonesia. Both species together are sister to another lineage of *S. sageneus*, which is widely distributed from Indonesia and western Australia to eastern Australia. Since *S. sageneus* is primarily distributed in Australian waters (type locality: western Australia), but also recorded from Bali, Indonesia, and other localities in the Eastern Indian Ocean, we suggest that the latter may represent the ‘true’ *S. sageneus*. In contrast, the genetically distinct (with 15.3–15.7% sequence divergence at *COI* from the others) and robustly resolved (by both ASAP and bPTP) lineage, represented by the HM422413 *S. sageneus* from Indonesia, could potentially represent a new species.

Within the *S. macrops/pacificus* clade, two morphospecies are present, with *S. pacificus* being a new species described in 2016 by Ho and colleagues, based on specimens collected in the western Pacific Ocean (Ho, Chen & Shao, 2016). This species is commonly found in bottom trawls off southwestern Taiwan at depths of around 100 m and is often confused with another *Synodus* species, *S. macrops*, which is also found in Taiwan and adjacent waters, due to their similar appearance (Ho, Chen & Shao, 2016). However, the two species exhibit subtle morphological differences: *S. macrops* has a black peritoneum, less distinct marks on the lateral body, and no bars on the dorsal and caudal fins, while *S. pacificus* has a white peritoneum, clear marks on the lateral body, and bars on the dorsal and caudal fins. We believe the polyphyletic nature of both species, as inferred in our *COI* tree, may be attributed to species misidentification and/or hidden species diversity. In our species delimitation analyses, ASAP clustered all individuals of both species into a single OTU, while bPTP separated them into six OTUs. After considering additional criteria, including morphological examination of available specimens, we propose resolving them as three OTUs or putative species: *S. pacificus*, the widely distributed *S. macrops*, and the Philippine-restricted *S.* cf. *macrops*. The genetic divergence among these three species ranges from 3.0% to 4.9% at the *COI* locus.

As to the *Synodus* sp. clade, it includes sequences from at least six nominal species: *S. macrocephalus*, *S. indicus*, *S. fuscus*, *S. doaki*, and *S. kaianus*. These are undoubtedly misidentifications, but due to the lack of voucher specimens for accurate species identification and morphological comparison, we tentatively infer them as a single putative species based on genetic diversity alone (genetic divergence <3.2% at *COI*) and refrain from assigning a species name pending further investigation (Fig. 5). Nevertheless, this undetermined “species” shows closer phylogenetic relationships to *S. indicus*, primarily distributed in the Indian Ocean, and *S. fuscus*, which is restricted to the Northwest Pacific, from Japan to Taiwan and China. The confused taxonomy of these closely related and morphologically similar species requires further resolution.

Finally, for the case of *Saurida undosquamis*, while ASAP inferred it as a single OTU, bPTP separated it into six. However, some of the bPTP OTUs displayed negligible inter-OTU divergence (<1%). We first merged these OTUs into a single one and resolved them all as three OTUs: two are sympatrically distributed in Australian waters from Queensland to western Australia, while the third is distributed from Japan to Taiwan and the South China Sea. Then, we further merged the two allopatric sister OTUs (Australian *vs.* Northwest Pacific plus South China Sea) based on the biogeographic criterion of Kekkonen & Hebert (2014) and limited genetic divergence (3.6% at *COI*). As a result, two putative species of *Saurida undosquamis* are tentatively proposed, with their distributions overlapping in waters off Queensland, Australia (Fig. 6).

#### Species diversity exploration

As a result of this study, 107 final inferred species were observed from the c.a. 60 lizardfish species in the two *COI* datasets, revealing an underestimated biodiversity. The inferred species clusters included some questionable taxa that are either undescribed or simply not well identified (*e.g.*, sequences downloaded from Genbank, Appleyard et al., 2025), and some independent evolutionary lineages were observed within a certain species.

According to our species delimitation and phylogenetic analyses, several recognized synodontid species were found to have two or more independent evolutionary lineages that are both genetically and geographically different. For instance, the lineage of *Synodus poeyi* distributed in the Caribbean Sea was separated from the lineage distributed in the Atlantic Ocean and Gulf of Mexico (Fig. 5). The two lineages of *S. poeyi* were noted by Frable et al. (2013). Within their study, the lineage occurring in the Caribbean Sea was regarded as a deeper water one. *Saurida nebulosa* (or the *S. nebulosa* complex) and *Saurida gracilis* (or the *S. nebulosa* complex) are even more intricate, comprising three to four genetically distinct species, deeply branching in the *COI* tree (Fig. 6), and generally separated by geography. Other examples of genetically less distinct sibling species were observed in the following morphospecies: *Synodus intermedius*, *S. saurus*, *Trachinocephalus myops*, *T. trachinus*, *Harpadon microchir*, *H. nehereus*, *Saurida brasiliensis* Norman, 1935, and *S. isarankurai* Shindo & Yamada, 1972. For instance, two inferred species of *Saurida brasiliensis* were identified, one occurring in the Gulf of Mexico and the other in Belize and Panama. *Harpadon microchir* exhibited a discrete lineage in Papua New Guinea, distinct from individuals found in Taiwan and Japan. Although all specimens within these lineages share the characteristic features of *H. microchir* (*e.g.*, the posterior tip of the pectoral fin does not reach the origin of the pelvic fin) (Nakabo, 2002), a detailed morphological examination is needed to confirm the validity of these two *Harpadon microchir* lineages. These findings suggest that allopatric speciation as a result of marine biogeographic barriers (Luiz et al., 2012; Hung, Russell & Chen, 2017; Lo et al., 2017) may be common among lizardfishes.

The genus *Trachinocephalus* was previously regarded as a monotypic genus containing only *Trachinocephalus myops*, a species with nearly circumtropical and subtropical distributions (Norman, 1935; Briggs, 1960; Anderson, Gehringer & Berry, 1966). With little difference in morphology, *T. trachinus* was regarded as a junior synonym of *T. myops* in Pacific region (Sulak, 1989). According to the taxonomic revision of the genus based on morphological and molecular evidences (Polanco, Acero & Betancur, 2016), *T. trachinus* is regarded as a valid species. With wider taxon sampling, the phylogenetic reconstruction in this study further reconfirmed *T. myops*, *T. trachinus* and the recently described *T. gauguini* as three valid species within the genus *Trachinocephalus*. In addition, the species delimitation results in the present study revealed two genetically and geographically different lineages in both *T. myops* and *T. trachinus*, indicating the presence of potential new species. This finding aligns with a recent investigation of *T. trachinus* along the Indian coast, which also indicated the existence of potential cryptic lineages in the Indian Ocean (Susanthi et al., 2023). Furthermore, our data shows *T. gauguini* occurs also in Madagascar (Fig. 5), demonstrating that the species has a much broader distribution than previously recognized.

Our results also challenge the validity of an extant species in the genus *Harpadon*. *Harpadon nudus* is a recently described species by Ganga, Thomas & Sukumaran (2015). Compared with other congeneric species, *H. nudus* was described as having a more slender body, shorter pectoral and pelvic fins, and body with very few scales. However, our species delimitation analyses indicated a conspecific relationship of *H. nudus* and *H. squamosus* (Fig. 6). Furthermore, only slight differences were found among the *COI* sequences between these two species (overall divergence: 0.9% ± 0.2%). Although Ganga, Thomas & Sukumaran (2015) provided molecular evidence with a UPGMA tree comparison with two congeners (*H. microchir* and *H. nehereus*), the genetic comparison of these two closely related species was not correctly made in the study. When proposing new species, it is essential to adopt an integrated approach that incorporates both traditional taxonomic methods and molecular data. However, molecular analyses should be conducted with caution, particularly when examining genetic variation among closely related species. All closely related species within the extant dataset (*e.g.*, GenBank or BOLD) should be included in the analysis. In the case of *H. nudus*, the validity of this species is questionable.

Despite the fact that many new lizardfish species have been described in recent years (Frable et al., 2013; Ganga, Thomas & Sukumaran, 2015; Russell, 2015; Ho, Chen & Shao, 2016; Allen, Erdmann & Peristiwady, 2017; Furuhashi, Russell & Motomura, 2023; Russell, Malay & Cabebe-Barnuevo, 2024; Furuhashi & Motomura, 2025a; Furuhashi & Motomura, 2025b), rather less attention has been paid to a comprehensive taxonomic revision of the family. Moreover, the observed number of putative species clusters within the species delimitation analysis in this study is still higher than that of the recognized species, indicating the taxonomy of the family is far from fully described and understood. During the course of data compiling, we found that the name, for instance *Saurida undosquamis*, has been confused with other species such as *S. gracilis*, *S. grandisquamis*, *S. macrolepis*, *S. lessepsianus*, and *S. elongata* in public data, which indicates a high frequency of misidentification or unresolved taxonomy. In this study, we can affirm the “true” species by the cluster that contained species from the type locality as well as reconfirmation based on morphology. In this case, a molecular approach provides valuable insights into some widespread species that otherwise may be misled by some symplesiomorphic characters. Additionally, *S. grandisquamis*, which was previously synonymized with *S. undosquamis* by Inoue & Nakabo (2006), appears to have independently evolved from “*S. undosquamis* species complex” based on the molecular phylogeny inferred in this study. We also confirm the species status of *S. umeyoshii, S. longimanus, and S. macrolepis* within this complex, suggesting that these species have evolved independently despite their morphological similarities. Furthermore, we provide additional support for the validation of recently described species, including *Saurida fortis*, *S. weijeni*, and the resurrected *S. eso* and *S. elongata* (Furuhashi, Russell & Motomura, 2023; Russell, Malay & Cabebe-Barnuevo, 2024; Russell, Motomura & Furuhashi, 2025), while refining their distribution ranges. For instance, our data supports and extends the range outside of the East Asian region of *S. fortis* (Furuhashi, Seah & Motomura, 2025) and also *S. umeyoshii* (Furuhashi & Motomura, 2025c). However, several inferred species remain unidentified or represent potential new species, highlighting the need for further revision of the taxonomy of lizardfishes.

#### Species diversity of lizardfishes in Taiwan and Papua New Guinea

Both Taiwan (at least the southern part) and Papua New Guinea are part of the Coral Triangle, the global center of marine fish diversity, and through the “*Tropical Deep-Sea Benthos*” biodiversity exploration program, we were able to collect a rich set of lizardfish specimens to document their regional species diversity (Table S1). Through this study, a total of 31 lizardfish species have been recorded in Taiwan, including 17 species from *Synodus*, one species from *Trachinocephalus*, two species from *Harpadon*, and 11 species from *Saurida*. In this study, we report the presence of eight species of *Synodus*, including *S. kaianus*, *S. pacificus*, *S. macrops*, *S. hoshinonis*; one species of *Trachinocephalus,* specifically *T. trachinus;* two species of *Harpadon,* namely *H. microchir* and *H. nehereus*; and ten species of *Saurida* including *S. gracilis* 1, *S. gracilis* 2, *S. micropectoralis*, *S. eso*, *S. elongata (ex. S. wanieso), S.* cf. *isarankurai*, *S. undosquamis*, *S.* sp. (bigeyes), *S. fortis*, and *S. macrolepis*, as occurring in Taiwanese waters. All except *Saurida eso* and one unidentified *Saurida species* have been previously reported. Following *Russell, Motomura & Furuhashi (2025)*, two species previously recognized in Taiwan, *Saurida eso* is now considered valid (resurrected from its previous synonymy with *S. elongata*); while *S. wanieso* is now synonymized with *S. elongata*. Through further morphological examination, we also observed the presence of one species with unique morphology (characterized by large eyes) and another species morphologically resembling *S. isarankurai*. These findings suggest that further detailed taxonomic investigations of *Saurida* species occurring in Taiwan is needed.

Additionally, *Synodus taiwanensis* Chen, Ho & Shao, 2007 and *Synodus tectus* Cressey, 1981 (Cressey, 1981) were previously recorded in Taiwan. However, our *COI* analyses revealed that sequences associated with these two names are nested within the *S. hoshinonis* clade, with minimal genetic differentiation. Examination of the voucher specimens of these two species showed no significant differences in their morphology or coloration (R Furuhashi, 2025, pers. Comm.), and we therefore propose that both names should be considered junior synonyms of *S. hoshinonis*. These two “species”, along with “*Saurida wanieso*” (which has been synonymized), should be excluded from the list of lizardfish species occurring in Taiwan.

On the other side, a total of 11 lizardfish species have previously been recorded in Papua New Guinean waters, including six species of *Synodus*, two of *Trachinocephalus*, and three of *Saurida* (Fricke et al., 2014; Fricke et al., 2019). In this study, over 55 specimens were collected during three of our expeditions to Papua New Guinea. Species delimitation analyses of these specimens, combined with data from online databases, revealed the presence of 14 species—representing four new records (*Harpadon aff. microchir*, *Synodus kaianus*, *S. pacificus*, and *Saurida umeyoshii*) and five putatively undescribed species (*Synodus* PNG1–5). These findings highlight the remarkably high diversity of deep-water lizardfishes in the region. Among these, *Harpadon* aff. *microchir* was remarkably collected at depths ranging from 470 to 985 meters—surpassing the previously deepest known record for a *Harpadon* species, which was 801 m for *H. erythraeus* (Klausewitz, 1983). Morphologically, the specimens resemble *H. microchir*, a species typically found along the continental shelf edge at depths of 400–600 m only. However, the Papua New Guinean specimens are genetically and ecologically distinct, suggesting they may represent a separate, undescribed species.

## Conclusions

This study investigated the molecular systematics of lizardfishes and provided the first reliable phylogeny, based on the combination of the mitochondrial and nuclear gene markers. Species diversity was also evaluated based on the comprehensive *COI* datasets, especially for the species distributed in the IWP region.

The results of the high-level phylogenetic analysis showed that the lizardfishes were not resolved as a monophyletic group. Instead, the two previously defined subfamilies, Synodontinae (containing *Synodus* and *Trachinocephalus*) and Harpadontinae (containing *Saurida* and *Harpadon*), were supported as distinct clades. Consequently, we propose elevating these two subfamilies to family-level taxonomic status, supported by evidence from both morphological and molecular approaches. The divergence times of the two newly defined families were also estimated in this study. *Synodontidae* appeared to have an earlier origin during the Late Cretaceous, while the origin of *Harpadontidae* was estimated to be in the Eocene.

At the species level, the *COI* gene data were explored and revealed a higher number of inferred species compared to the recognized species included in the study. This result suggests the presence of several potential new species, including five identified in Papua New Guinean waters, as well as others in Taiwan, Australia, and the western Indian Ocean. Further taxonomic revision of the families is therefore required.

Although the analyses did not include all recognized lizardfish species globally due to limitations in sample collection, the present study provides a framework for future generic and species-level taxonomic revision, as well as phylogenetic, and biogeographic research. We also offer valuable genetic references with accurate species identification for subsequent DNA (or eDNA) barcoding/meta-barcoding analyses, which are crucial for documenting the biodiversity of lizardfishes.

##  Supplemental Information

10.7717/peerj.20735/supp-1 Supplemental Information 1 List of gene sequences obtained in this study, including species names, sample IDs, voucher IDs, sample locations, and GenBank accession numbers for each sequence

10.7717/peerj.20735/supp-2 Supplemental Information 2 Primers used in this study Abbrebiations of genes: RAG1, recombination activation gene 1; RH: Rhodopsin; ZIC1: zic family member 1; ENC1: ectodermal-neural cortex 1; COI, mitochondrial cytochrome c oxidase subunit 1; 12S: 12S ribosomal RNA gene. Reverse primers in italics.

10.7717/peerj.20735/supp-3 Supplemental Information 3 The best-fit model for each partition determined using ModelTest-NG

10.7717/peerj.20735/supp-4 Supplemental Information 4 Species list of Genbank sequences included in the high-level phylogenetic analyses, including the species name, Sample ID, family name (newly defined) and source of each sequences

10.7717/peerj.20735/supp-5 Supplemental Information 5 Previously proposed classifications in relation to the traditional family Synodontidae (Partial) Author names and proposed years appear in bold.

10.7717/peerj.20735/supp-6 Supplemental Information 6 Overview of datasets used in this study Arrows represent the sources and flow of data utilized in the analyses.

10.7717/peerj.20735/supp-7 Supplemental Information 7 Inferred ML *COI* tree (*Synodus*)

10.7717/peerj.20735/supp-8 Supplemental Information 8 Inferred ML COI tree (Trachinocephalus)

10.7717/peerj.20735/supp-9 Supplemental Information 9 Inferred ML COI tree (Saurida)

10.7717/peerj.20735/supp-10 Supplemental Information 10 Inferred ML COI tree (Harpadon)

10.7717/peerj.20735/supp-11 Supplemental Information 11 Newly generated DNA sequences of this study

## Additional Information and Declarations

### Field Study Permissions

The following information was supplied relating to field study approvals (*i.e.*, approving body and any reference numbers): The permits of New Caledonian expeditions were issued by the respected authorities in the New Caledonia with inventory nos. APA_NCPS_2016_012 and ZEE APA-NC01. The permits of BIOMAGLO were issued by the ”Terres Australes et Antarctiques Françaises” (TAAF), Mayotte, and Comorres. The SAYA expedition is part of the 2e leg of the “Indian Ocean 2022” survey operated by MonacoExplorations under MoU MNHN-JMA 1176-22. The Papua New Guinea expeditions were also supported from Papua New Guinea’s National Fisheries Authority and conducted under a MoU with the University of Papua New Guinea (UPNG), and with a permit from the Papua New Guinea Department of Environment and Conservation (DEC). Expeditions conducted in Taiwanese waters were approved by the National Science and Technology Council, Taiwan (NSTC) (102-2923-B-002-001-MY3, 110-2611-M-002-013, 113-2611-M-002-014).

### Data Availability

The following information was supplied regarding data availability: The molecular data are available at GenBank: PV872621–PV872834 (*COI*); PV879956–PV879979 (*RAG1*); PV879980–PV880002 (*RH*); PV880003–PV880035 (*ENC1*); PV880036–PV880073 (*ZIC1*); PV882391–PV882433 (*12S*).
