# Supplementary material for: Beyond the sandy bottom: evolutionary and taxonomic insights into lizardfishes (Teleostei: Aulopiformes)
Source: PeerJ. 2026 Mar 6;14:e20735. doi: 10.7717/peerj.20735 (PMC12970317; doi:10.7717/peerj.20735)
Supplement: Supplemental Information 5 — Author names and proposed years appear in bold. [file peerj-14-20735-s005.pdf]

Gosline et al. (1966)

Order Iniomi
- Myctophoidea
  - Aulopidae
  - Bathysauridae
  - Synodontidae
  - Harpadontidae
  - Bathypteroidae
  - Ipnopidae
  - Chlorophthalmidae
  - Notosudidae (=Scopelosauridae)
  - Myctophidae
  - Neoscopelidae
- Alepisauroidea
  - Paralepididae
  - Omosudidae
  - Alepisauridae
  - Anotopteridae
  - Evermannellidae
  - Scopelarchidae

Rosen (1973)

Order Aulopiformes, new name
- Suborder Aulopoidei, new name
  - Aulopidae
  - Bathysauridae
  - Ipnopidae
  - Chlorophthalmidae
  - Notosudidae (=Scopelosauridae)
- Suborder Alepisauroidei
  - Superfamily Synodontoidea, new usage
    - Synodontidae
    - Harpadontidae
    - Giganturidae
  - Superfamily Alepisauroidea
    - Paralepididae
    - Omosudidae
    - Alepisauridae
    - Anotopteridae
    - Evermannellidae
    - Scopelarchidae

Sulak (1977)

Benthic Myctophiformes:
- Aulopidae (*Aulopus, Hime Latropiscus*)
- Synodontidae
  - Subfamily Harpadontinae (*Harpadon, Saurida*)
  - Subfamily Bathysaurinae (*Bathysaurus*)
  - Subfamily Synodontinae (*Synodus, Trachinocephalus*)
- Chlorophthalmidae
  - Subfamily Chlorophthalminae (*Chlorophthalmus, Parasudis, Bathysauropsis*)
  - Subfamily Ipnopinae
    - Tribe Ipnopini (*Ipnops*)
    - Tribe Bathypteroinl (*Bathypterois*)
    - Tribe Bathymicropini (*Bathymlcrops, Bathytyphlops*)

R. K. Johnson (1982)

Myctophiformes:
- Aulopoids
  - Aulopidae
- Myctophoids + Chlorophthalmoids
  - Myctophoids
    - Myctophidae
    - Neoscopelidae
  - Chlorophthalmoids
    - Notosudidae
    - Scopelarchidae
    - Chlorophthalmidae
    - Ipnopidae
- Synodontoids + Alepisauroids
  - Synodontoids
    - Synodontidae
    - Harpadontldae
    - Bathysauridae
  - Alepisauroids
    - Paralepldidae
    - Anotopteridae
    - Evermannellidae
    - Omosudidae
    - Alepisauridae

Figure S1. Previously proposed classifications in relation to the traditional family Synodontidae (Partial). Author’s name and proposed years were shown in **Bold**.

Baldwin & Johnson (1996)
Order Aulopiformes
  Suborder Synodontoidei
    Family Aulopidae (*Aulopus*)
    Family Pseudotrichonotidae (*Pseudotrichonotus*)
    Family Synodontidae (*Harpadon, Saurida, Synodus, Trachinocephalus*)
  Suborder Chlorophthalmoidei
    Family Chlorophthalmidae (*Chlorophthalmus, Parasudis*)
    Bathysauropsis (*B. gracilis, B. malayanus*)
    Family Notosudidae (*Ahliesaurus, Luciosudis, Scopelosaurus*)
    Family Ipnopidae (*Bathymicrops, Bathypterois, Bathytyphlops, Ipnops*)
  Suborder Alepisauroidei
    Family Alepisauridae (*Alepisaurus, Omosudis*)
    Family Paralepididae
    Family Evermannellidae (*Coccorella, Evermannella, Odontostomops*)
    Family Scopelarchidae
  Suborder Giganturoidei
    Bathysauroides gigas
    Family Bathysauridae (*Bathysaurus*)
    Family Giganturidae (*Gigantura*)

Sato & Nakabo (2002)
Order Aulopiformes
  Suborder Synodontoidei
    Family Paraulopidae (Paraulopus)
    Family Aulopidae (Aulopus)
    Family Pseudotrichonotidae (Pseudotrichonotus)
    Family Synodontidae (Harpadon, Saurida, Synodus, Trachinocephalus)
  Suborder Chlorophthalmoidei
    Family Bathysauroididae (Bathysauroides)
    Family Chlorophthalmidae (Chlorophthalmus, Parasudis)
    Family Bathysauropsidae (Bathysauropsis)
    Family Notosudidae (Ahliesaurus, Luciosudis, Scopelosaurus)
    Family Ipnopidae (Bathymicrops, Bathypterois, Bathytyphlops, Ipnops)
  Suborder Alepisauroidei
    Family Alepisauridae (Alepisaurus, Omosudis)
    Family Paralepididae
    Family Evermannellidae (Coccorella, Evermannella, Odontostomops)
    Family Scopelarchidae
  Suborder Giganturoidei
    Family Bathysauridae (Bathysaurus)
    Family Giganturidae (Gigantura)

Davis (2010)
Order Aulopiformes
  Suborder Aulopoidei
    Family Synodontidae (*Synodus, Trachinocephalus, Harpadon, Saurida*)
    Family Aulopidae (*Aulopus*)
    Family Pseudotrichonotidae (*Pseudotrichonotus*)
  Suborder Paraulopoidei
    Family Paraulopidae (*Paraulopus*)
  Suborder Alepisauroidei
    Superfamily Ipnopoidea
      Epifamily Giganturoidae
        Family Giganturidae (*Gigantura*)
        Family Bathysauridae (*Bathysaurus*)
        Family Bathysauroididae (*Bathysauroides*)
      Epifamily Ipnopoidae
        Family Bathysauropsidae (*Bathysauropsis*)
        Family Ipnopidae (*Bathypterois, Ipnops, Bathymicrops, Bathytyphlops*)
    Superfamily Chlorophthalmoidea
        Family Chlorophthalmidae (*Chlorophthalmus, Parasudis*)
    Superfamily Notosudoidea
        Family Notosudidae (*Scopelosaurus, Ahliesaurus, Luciosudis*)
    Superfamily Alepisauroidea sensu nov.
        Family Scopelarchidae (*Benthalbella, Rosenblattichthys, Scopelarchus, Scopelarchoides*)
        Family Evermannellidae (*Odontostomops, Coccorella, Evermannella*)
        Family Sudidae (*Sudis*)
        Family Alepisauridae sensu nov. (*Anotopterus, Magnisudis, Omosudis, Alepisaurus*)
        Family Paralepididae sensu nov. (*Macroparalepis, Paralepis, Arctozenus, Stemonosudis, Lestidiops, Uncisudis, Lestrolepis, Lestidium, Dolichosudis*)

Figure S1. (continued)
