# Supplementary material for: Beyond the sandy bottom: evolutionary and taxonomic insights into lizardfishes (Teleostei: Aulopiformes)
Source: PeerJ. 2026 Mar 6;14:e20735. doi: 10.7717/peerj.20735 (PMC12970317; doi:10.7717/peerj.20735)
Supplement: Supplemental Information 6 — Arrows represent the sources and flow of data utilized in the analyses. [file peerj-14-20735-s006.pdf]

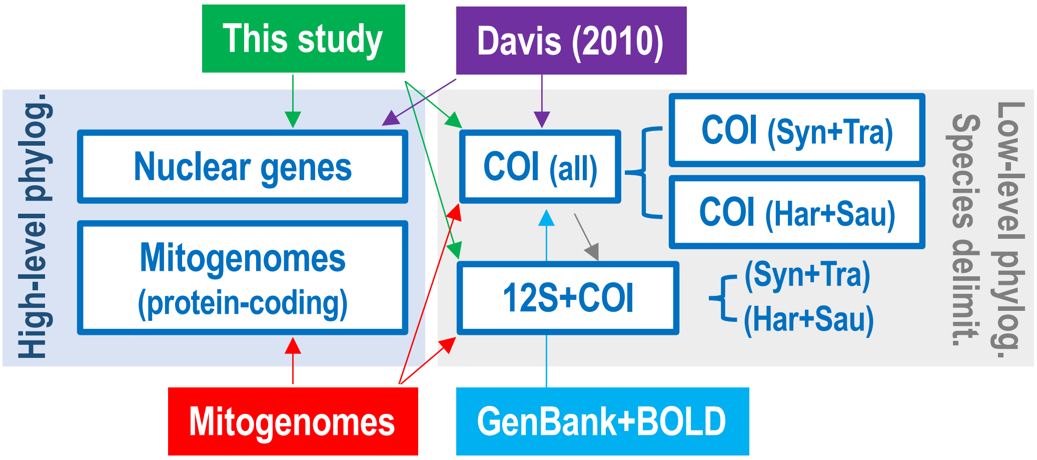


**Figure S2.** Overview of datasets used in this study. Arrows represent the sources and flow of data utilized in the analyses.
