# Supplementary figures and images for: Beyond the sandy bottom: evolutionary and taxonomic insights into lizardfishes (Teleostei: Aulopiformes)
Source: PeerJ. 2026 Mar 6;14:e20735. doi: 10.7717/peerj.20735 (PMC12970317; doi:10.7717/peerj.20735)

**Figure S3.** Inferred ML *COI* tree (*Synodus*)

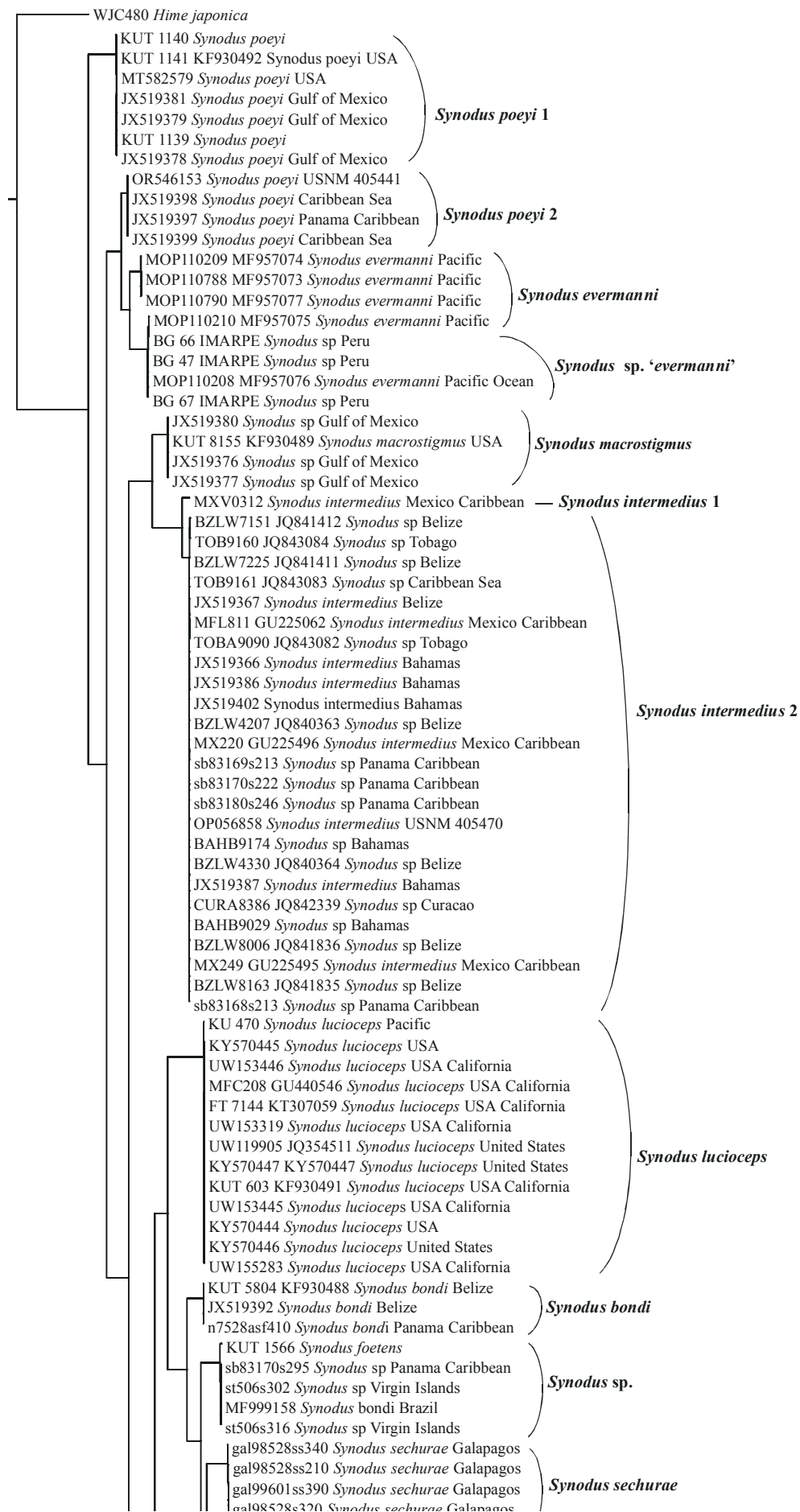

Supplement: Supplemental Information 7 [file peerj-14-20735-s007.pdf]

Figure S4. Inferred ML *COI* tree (*Trachinocephalus*)

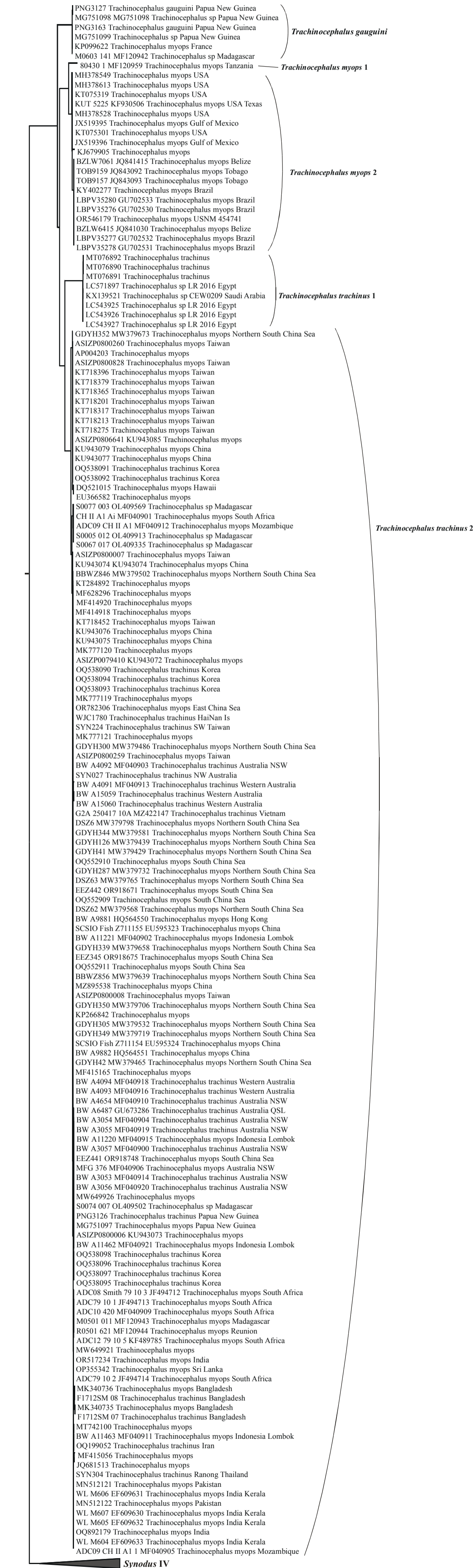

Supplement: Supplemental Information 8 [file peerj-14-20735-s008.pdf]

Figure S6. Inferred ML *COI* tree (*Harpadon*)

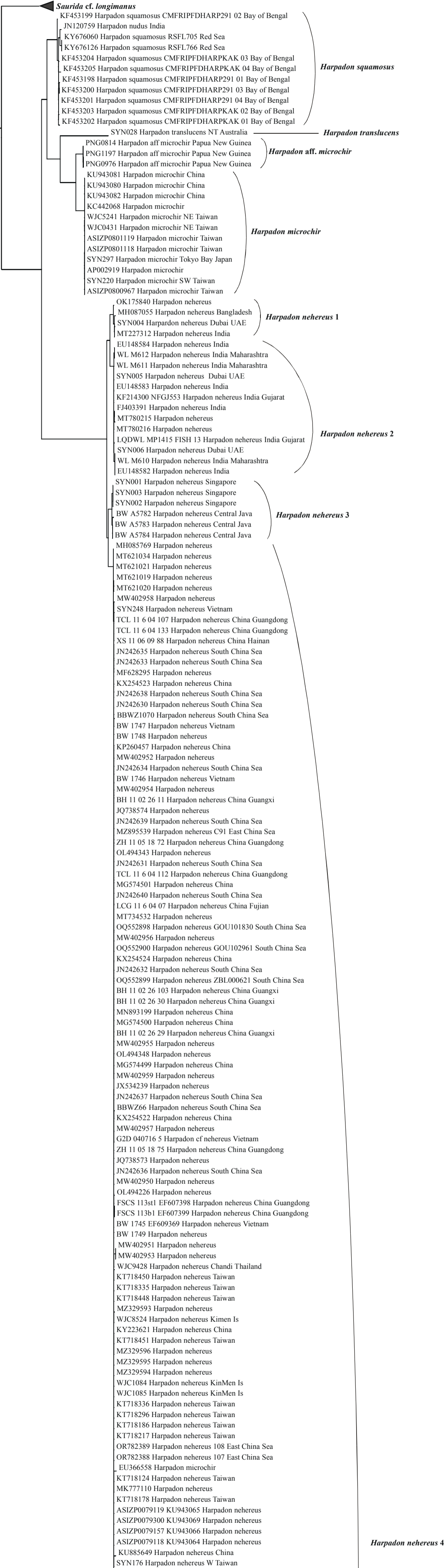

Supplement: Supplemental Information 10 [file peerj-14-20735-s010.pdf]
