# Supplementary material for: Beyond the sandy bottom: evolutionary and taxonomic insights into lizardfishes (Teleostei: Aulopiformes)
Source: PeerJ. 2026 Mar 6;14:e20735. doi: 10.7717/peerj.20735 (PMC12970317; doi:10.7717/peerj.20735)

Figure S5. Inferred ML *COI* tree (*Saurida*)

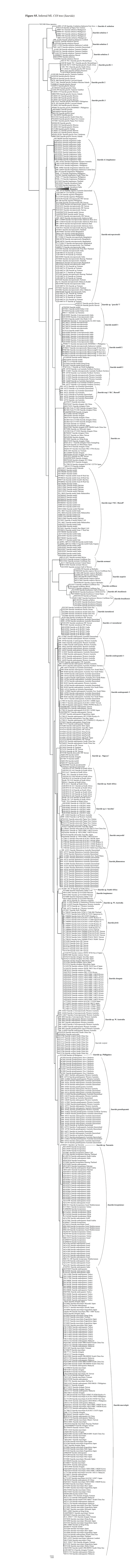

Supplement: Supplemental Information 9 [file peerj-14-20735-s009.pdf]
